# Human induced mesenchymal stem cells display increased sensitivity to matrix stiffness

**DOI:** 10.1038/s41598-022-12143-2

**Published:** 2022-05-19

**Authors:** Kirstene A. Gultian, Roshni Gandhi, Khushi Sarin, Martina Sladkova-Faure, Matthew Zimmer, Giuseppe Maria de Peppo, Sebastián L. Vega

**Affiliations:** 1 Department of Biomedical Engineering, Rowan University, Glassboro, NJ 08028 USA; 2 The New York Stem Cell Foundation Research Institute, New York, NY 10019 USA

**Keywords:** Stem cells, Mesenchymal stem cells, Pluripotent stem cells

## Abstract

The clinical translation of mesenchymal stem cells (MSCs) is limited by population heterogeneity and inconsistent responses to engineered signals. Specifically, the extent in which MSCs respond to mechanical cues varies significantly across MSC lines. Although induced pluripotent stem cells (iPSCs) have recently emerged as a novel cell source for creating highly homogeneous MSC (iMSC) lines, cellular mechanosensing of iMSCs on engineered materials with defined mechanics is not well understood. Here, we tested the mechanosensing properties of three human iMSC lines derived from iPSCs generated using a fully automated platform. Stiffness-driven changes in morphology were comparable between MSCs and iMSCs cultured atop hydrogels of different stiffness. However, contrary to tissue derived MSCs, no significant changes in iMSC morphology were observed between iMSC lines atop different stiffness hydrogels, demonstrating a consistent response to mechanical signals. Further, stiffness-driven changes in mechanosensitive biomarkers were more pronounced in iMSCs than MSCs, which shows that iMSCs are more adaptive and responsive to mechanical cues than MSCs. This study reports that iMSCs are a promising stem cell source for basic and applied research due to their homogeneity and high sensitivity to engineered mechanical signals.

## Introduction

Mesenchymal stem cells (MSCs) are non-hematopoietic cells capable of differentiating into cells that produce various mesodermal tissues, including osteoblasts, adipocytes, and chondrocytes^1^. MSCs are present in numerous stem cell niches including bone marrow and adipose tissue and can be expanded in vitro by plating onto tissue culture polystyrene (TCPS), which causes them to adhere, adopt a spindle-like shape, and proliferate into fibroblastic colony-forming units^2^. Owing to their unique properties and ease of expansion, MSCs have been extensively studied and used in numerous clinical trials for the treatment of various medical disorders^3,4^.

In vivo, the multifunctional phenotype of MSCs is regulated by chemical and physical cues of the tissue microenvironment^5^, which influence numerous functions including migration, differentiation, and paracrine signaling^6^. To regulate stem cell behavior outside of the body, biomaterials are used to recapitulate specific properties of tissue microenvironments. For example, ECM elasticity and tissue-level stiffness are strong drivers of cellular mechanosensing and phenotypic commitment in vitro^7^. Engler et al. showed that MSCs atop soft hydrogels that mimic the stiffness of brain tissue express neuronal biomarkers, whereas MSCs on rigid substrates produce osteocalcin, a bone tissue-specific protein secreted by osteoblasts^7^. On a molecular level, MSC mechanosensing is led by several mechano-transducer proteins that collectively induce changes in focal adhesion maturation^8,9^, cytoskeletal contractility/alignment^10,11^, and nuclear Yes-associated protein (YAP) localization^12^.

Although engineered mechanical cues can regulate MSC mechanosensing in vitro, MSC populations are heterogeneous^13^, and donor variability between MSC lines derived from adult tissues is significant, resulting in inconsistent responses to engineered signals^14^. These challenges limit the possibility of manufacturing high quality, homogeneous MSC lines in large numbers needed for basic research and stem cell-based therapies^15^. When successfully reprogrammed, induced pluripotent stem cells (iPSCs) reset any possible mechanical memory that could bias their response to engineered materials by erasing historical epigenetic, transcriptional, and non-genetic information^16,17^. Indeed, iPSCs have recently been differentiated into functional MSCs (iMSCs), displaying phenotypic similarities with tissue derived MSCs^18,19^. However, there are only a limited number of studies that have explored the use of iMSCs^20^, and the effects of mechanical signals on iMSC mechanosensing is not well understood. In this study we sought to test two hypotheses: (1) iMSCs “feel” mechanical properties resulting in changes in morphology and intracellular mechanosensitive protein organization (focal adhesion maturation, actin alignment, YAP localization) comparable to MSCs, and (2) iMSC mechanosensitivity to substrate stiffness is more reproducible within iMSC populations and across iMSC lines as a consequence of the reprogramming process, which erases mechanical memories acquired during development and in response to engineered biophysical cues.

To test our hypotheses, we evaluated cell-material interactions of three human iMSC lines derived from iPSCs generated using a robotic, fully automated platform, which results in the production of highly reproducible iMSC lines^21^. We report that iMSCs are more responsive to matrix stiffness than human MSCs derived from adult tissues, and that stiffness-mediated changes in cellular mechanosensing are more consistent across different iMSC lines.

## Results and discussion

### Automated manufacturing of iPSC lines enables consistent production of iMSCs

**Figure 1:**
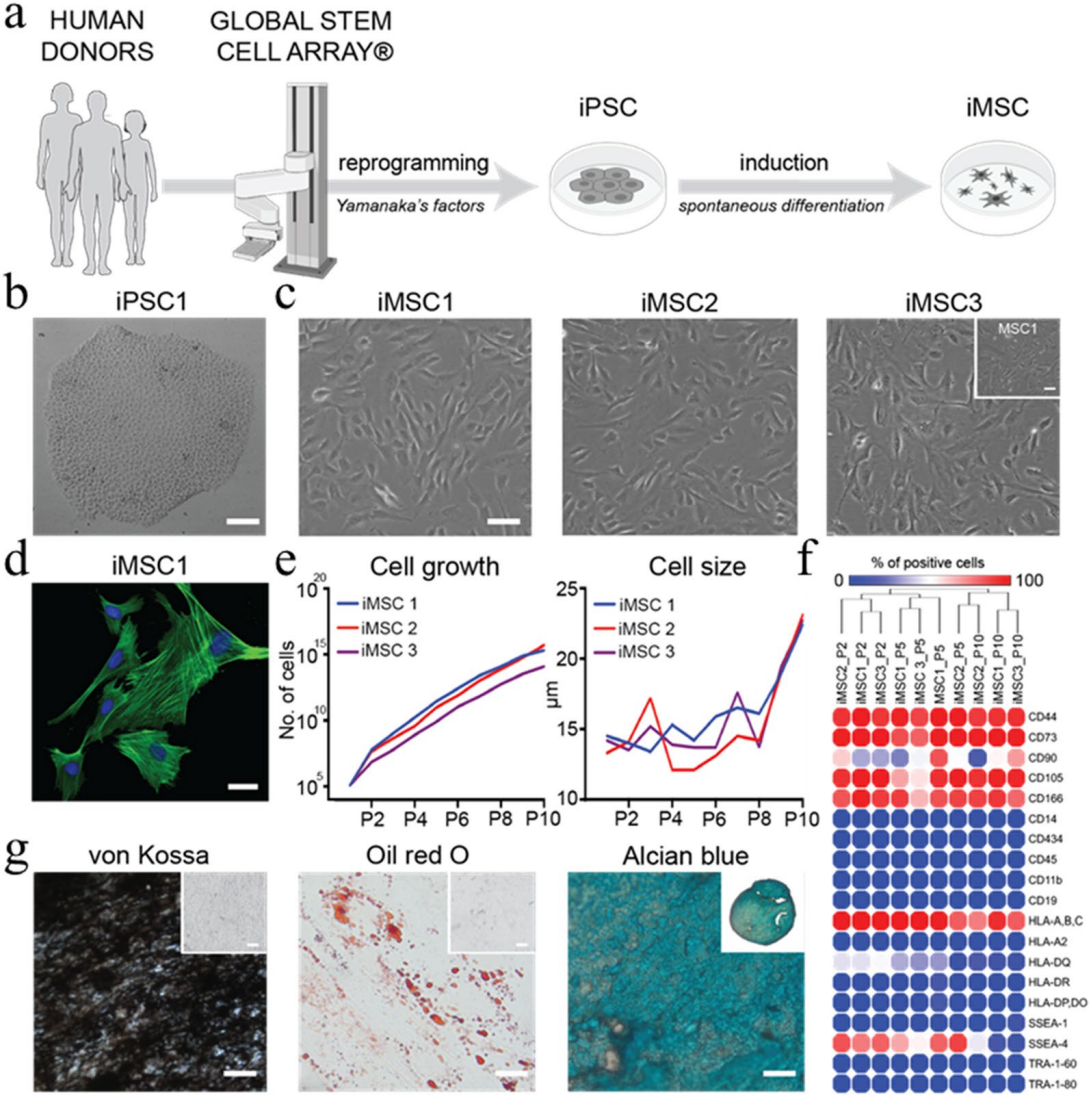
Derivation and characterization of human iMSC lines. (**a**) Schematic of automated reprogramming of human fibroblasts into iPSC lines using a NYSCF Global Stem Cell Array®. (**b**) Representative brightfield image of iPSC colony generated using automated platform. Scale bar, 100 µm. (**c**) Representative brightfield images of iMSC lines derived from three different donors at passage 5. Inset shows commercially available bone marrow derived MSCs at passage 5. Scale bar, 50 µm. (**d**) Representative confocal image of iMSCs stained for actin (green) and nuclei (blue). Scale bar, 20 µm. (**e**) Plots of cell growth and average cell length for 3 iMSC lines over 10 passages. (**f**) Hierarchical clustering of surface marker screening data for 3 iMSC lines at passage 2, 5, and 10 and MSCs at passage 5. (**g**) Representative brightfield images of iMSCs (line 1) differentiated towards osteogenic (von Kossa), adipogenic (Oil Red O), and chondrogenic (Alcian blue) tissues. Insets represent negative controls and full-size cartilage spheroids. Scale bar, 20 µm.

Manual production of human iPSC lines is time consuming and can result in significant line-to-line variability. To enable consistent production of high-quality and highly reproducible iMSC lines, fibroblasts from human skin biopsies were reprogrammed into stem cells using the NYSCF Global Stem Cell Array®, a modular, robotic platform for high-throughput production, maintenance, and differentiation of iPSCs (Fig. 1a). Three iPSC lines were manufactured and validated by their positive OCT4 and TRA-1-60 expression (Supplementary Fig. S1), visual confirmation of colonies on TCPS (Fig. 1b), and additional quality control metrics including sterility, karyotyping, genotyping, pluripotency expression profile, and differentiation capacity^21^.

Manufactured iMSC lines adhere to TCPS, feature a spindle-like morphology (Fig. 1c), and exhibit pronounced actin fibers (Fig. 1d). These morphological traits are comparable to human MSCs isolated from adult tissues and cultured atop TCPS substrates. The in vitro expansion rate (Fig. 1e, left) was consistent across all iMSC lines and iMSCs divided faster than adult MSCs in accordance with previously published data^22,23^. Interestingly, in vitro expansion over ten passages also results in a reproducible and progressive increase in average cellular length, which is a phenomenon also observed in tissue derived MSCs (Fig. 1e, right).

All iMSC lines are negative for the pluripotency markers OCT4 and TRA-1-60, confirming that they do not dedifferentiate into iPSCs or iPSC-like cells after at least ten passages on TCPS (Supplementary Fig. S2). A cell surface marker screening panel also confirmed that iMSCs are negative for other typical pluripotency and hematopoietic markers (Fig. 1f, blue circles). Importantly, iMSCs express mesenchymal markers including CD44, CD73, CD90, CD105, and CD166 similarly to MSCs isolated from adult tissues (Fig. 1f, red circles and Supplementary Table S1)^24^. iMSCs also express SSEA-4 at early passages, as seen in multipotent subpopulations of human MSCs isolated from bone marrow and other tissues^25^. The cell surface marker screening panel also shows that the iMSC lines express lower levels of human leukocyte antigens (HLA) class II, which suggests iMSCs are more immunoprivileged than adult MSCs^20,26^. Notably, iMSCs express more integrin alpha 2 (CD49b), integrin alpha 3 (CD49c), and integrin alpha 4 (CD49d) than adult MSCs (Table S1), and studies have shown that these integrins play a central role in mechanotransduction^27,28^. Thus, the higher expression of these integrins could enhance sensitivity to matrix stiffness.

In addition to these phenotypic features, the ability to differentiate towards osteogenic, adipogenic, and chondrogenic lineages in vitro is a hallmark trait of MSCs^1^. By exposing iMSCs to soluble differentiation factors, we demonstrate that iMSCs give rise to osteogenic, adipogenic, and chondrogenic lineages as evidenced by von Kossa, Oil Red O, and Alcian blue staining, respectively (Fig. 1g and Supplementary Fig. S3). Taken together, these findings confirm that iMSCs are phenotypically similar and possess the differentiation capacity of human MSCs.

### Stiffness-driven changes in iMSC morphology are consistent across multiple iMSC lines

While there are many macromers that can be used to synthesize hydrogels, hyaluronic acid (HA) hydrogels are highly biocompatible and amenable to extensive biochemical and biophysical modifications^29^. Additionally, HA is a commonly used macromer to form hydrogels that regulate MSC shape and mechanosensing^30,31^. To investigate the effects of stiffness on iMSC morphology, we were interested in culturing iMSCs on HA hydrogels that spanned a physiologic range in mechanics^7,12,30,31^. Though changes in MSC shape are seen across hydrogels as soft as 0.1 kPa and stiff as 40 kPa^12^, Cosgrove et al. found large changes on MSC morphology, nuclear YAP localization, actin anisotropy, and focal adhesion maturation between MSCs on soft (5 kPa), intermediate (10 kPa), and stiff (20 kPa) HA hydrogels^30^. Thus, we sought to synthesize HA hydrogels with a stiffness range of 5–20 kPa to study the effects of mechanics on iMSC shape and mechanotransduction.

Hydrogels were formed by photocrosslinking norbornene groups in hyaluronic acid (HA) macromers with thiols in DTT crosslinkers as previously reported^32^. The amount of macromer (3 wt%) was kept constant and Low (5.19 ± 1.04 kPa), Med (9.58 ± 0.98 kPa), and High (19.27 ± 2.41 kPa) matrix stiffness hydrogels were formed by varying the amount of crosslinker added (Supplementary Fig. S4). It is important to maintain the amount of HA constant since MSCs interact with HA via surface receptors including CD44 and CD168^33^. To promote cell adhesion, thiolated RGD peptides were coupled to the macromer backbone using a procedure described in the supplemental methods section (Supplementary Fig. S4), and ^1^H NMR was used to confirm HA modifications (Supplementary Fig. S5).

**Figure 2:**
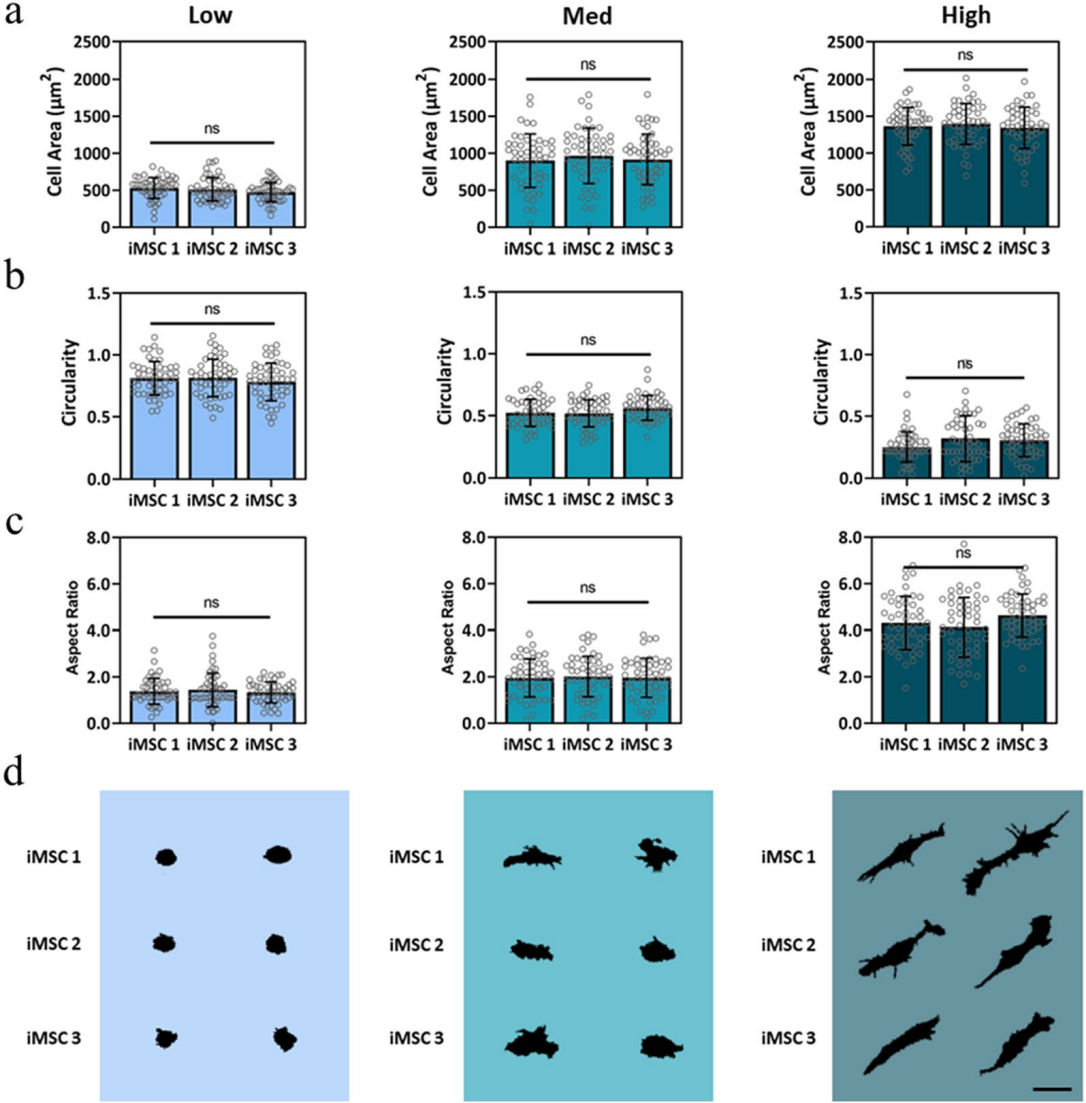
Effects of matrix stiffness on iMSC morphology. Single-cell image analysis was performed to attain iMSC (**a**) cell area, (**b**) circularity, and (**c**) aspect ratio for three iMSC lines cultured on Low, Med, and High stiffness hydrogels. (**d**) Representative single-cell silhouettes of iMSCs cultured atop Low, Med, and High hydrogels shown. Scale bar, 50 µm. Bar graphs represent the mean and error bars represent standard deviation; n > 50 cells per group, *n.s.* not significant.

To evaluate the effects of stiffness on iMSC morphology, three different iMSC lines were cultured atop Low, Med, and High stiffness hydrogels. After three days in culture, all iMSC lines displayed comparable stiffness-mediated changes in morphology. Cell area for iMSCs on Low, Med, and High stiffness hydrogels was 530 ± 141, 900 ± 360, and 1400 ± 250 µm^2^, respectively (Fig. 2a). Although average cell area values were consistent with data of MSCs cultured on hydrogels of comparable stiffness, there were significant differences in area across different MSC lines (Supplementary Fig. S6). In contrast, low heterogeneity was observed within and across iMSC lines for iMSCs cultured on Low, Med, and High stiffness hydrogels (Fig. 2a). Next, we evaluated circularity since MSC roundness decreases with increasing stiffness on 2D substrates^31^. Analogous to MSCs, iMSC circularity decreased with increasing stiffness, with circularity values ranging from 0.81 ± 0.14 for iMSCs on Low to 0.24 ± 0.12 for iMSCs on High stiffness hydrogels (Fig. 2b).

Aspect ratio indicates cellular elongation, and iMSC aspect ratio increased with increasing stiffness, with aspect ratio values ranging from 1.32 ± 0.51 for iMSCs on Low to 4.31 ± 0.81 on High stiffness hydrogels (Fig. 2c). Although iMSC and MSC stiffness-mediated circularity and aspect ratio trends are consistent, there is significant variability in MSC circularity (Supplementary Fig. S7) and aspect ratio (Supplementary Fig. S8) values. Representative images of single-cell silhouettes show observable differences in morphology in different stiffness groups but show no discernable differences across iMSC lines cultured on Low, Med, and High stiffness hydrogels (Fig. 2d). Taken together, these results support our hypotheses that stiffness-driven changes in iMSC morphology follow the same trend as MSCs and that iMSC morphology is highly consistent across iMSC lines.

### Mechanosensitive biomarkers of iMSCs are significantly impacted by matrix mechanics

**Figure 3:**
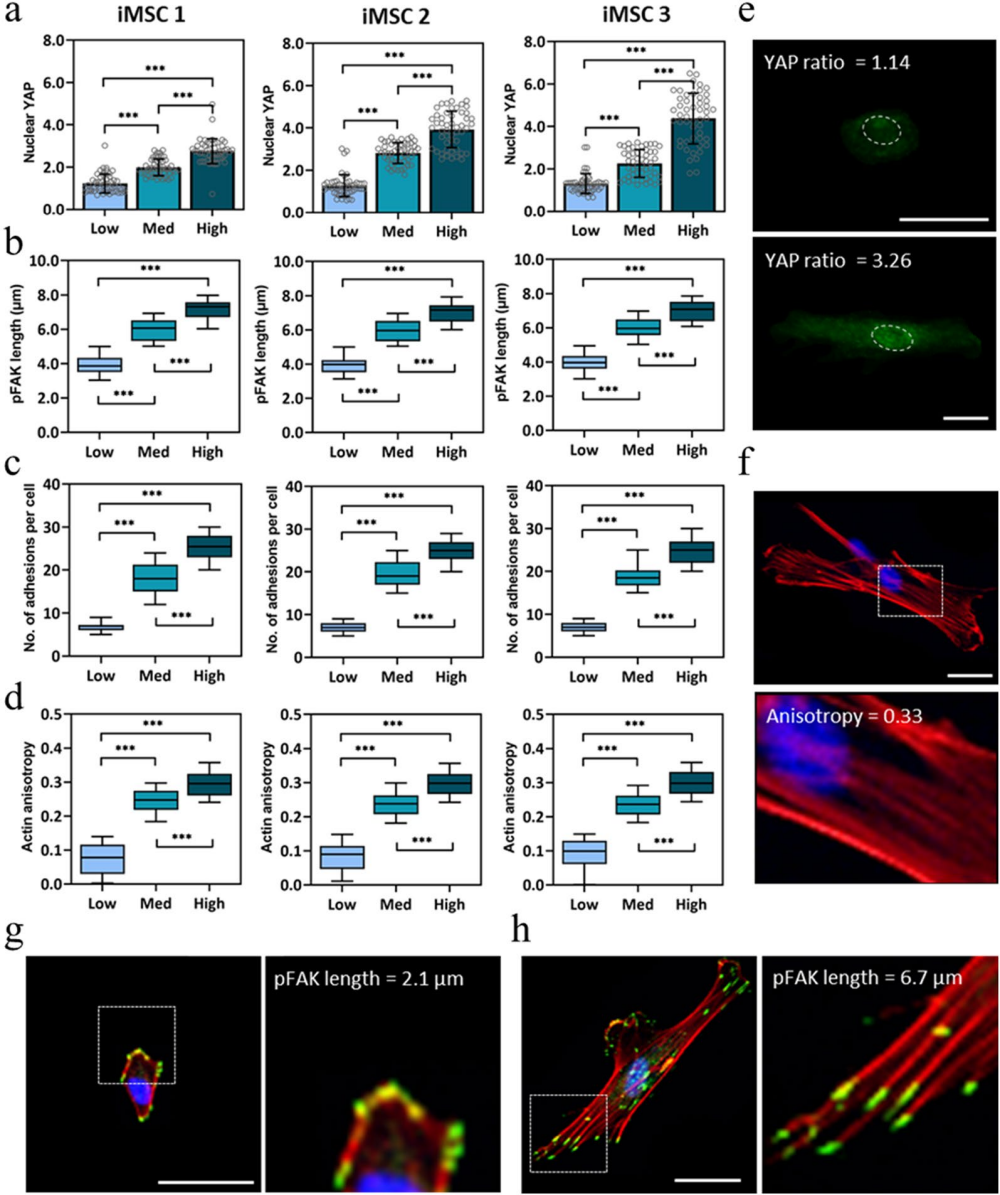
Mechanosensitivity of iMSC lines. Single-cell image analysis of (**a**) nuclear YAP localization, (**b**) pFAK length, (**c**) number of adhesions per cell, and (**d**) actin fiber anisotropy of iMSCs cultured atop Low, Med, and High stiffness hydrogels. (**e**) Representative quantifications of high and low nuclear YAP ratios (YAP, green; nucleus, white dashed oval). Scale bar, 50 µm. (**f**) Representative image and quantification of iMSC cultured atop Med stiffness hydrogel (red, actin; blue, nucleus). Scale bar, 50 µm. Representative image and quantification of iMSC cultured atop a (**g**) Low and (**h**) High stiffness hydrogel (green, pFAK; red, actin; blue, nucleus). Scale bar, 50 µm. Bar graphs represent the mean and error bars represent standard deviation. Box plots show 25/50/75th percentiles, whiskers show minimum/maximum; n > 50 cells per group, ***p < 0.001.

After demonstrating that iMSC morphology is highly consistent across donors and matrix stiffness groups, we evaluated stiffness-driven changes in iMSC mechanosensing. YAP acts as a nuclear relay of mechanical signals exerted by matrix stiffness and cell shape^12,31,34^. In MSCs, YAP is predominantly cytoplasmic in small and round cells and is nuclear in spread cells. After three days in culture, all iMSC lines displayed increasing nuclear YAP with increasing stiffness, with nuclear YAP values ranging from 1.22 ± 0.44 on Low to 2.76 ± 0.58 on High stiffness hydrogels (Fig. 3a). Although the stiffness-mediated trend in average nuclear YAP values is consistent with data of MSCs cultured on hydrogels of comparable stiffness^31^, the range in nuclear YAP values across stiffness groups is much larger for iMSCs than for MSCs (Supplementary Fig. S9).

Besides nuclear YAP localization, focal adhesion maturation and actin anisotropy give an insight to MSC mechanosensing^11,35^. Phosphorylated focal adhesion kinase (pFAK) is known to initialize at least two signaling pathways of MSC mechanosensing and plays an important role in controlling several cellular processes including cell spreading, migration, and focal adhesion maturation^36,37^. After three days in culture, all iMSC lines displayed an increase in focal adhesion maturation with increasing matrix stiffness. pFAK length for iMSCs on Low, Med, and High stiffness hydrogels was 3.94 ± 0.56, 5.98 ± 0.62, and 7.18 ± 0.56 µm, respectively (Fig. 3b). Number of adhesions per cell also increased with increasing stiffness, with an average of 6 ± 1 focal adhesions per cell on Low and 25 ± 3 on High stiffness hydrogels (Fig. 3c). Actin anisotropy is a measure of actin stress fiber alignment, and MSCs on stiff matrices (≥ 20 kPa) exhibit high cytoskeletal tension, resulting in anisotropic actin fibers^38^. Actin anisotropy of iMSCs also increased with increasing stiffness, and actin anisotropy values were consistent across all iMSC lines (Fig. 3d).

Average iMSC pFAK length values show low standard deviations within stiffness groups and a large range in pFAK lengths of iMSCs atop Low, Med, and High stiffness hydrogels. The average iMSC pFAK length is ~ 2 µm longer than for MSCs on the same stiffness conditions, and the standard deviations are lower for iMSCs on every stiffness group (Supplementary Fig. S10). Similarly, iMSC lines exhibit more adhesions per cell (~ 10 more adhesions) than MSCs for every stiffness group (Supplementary Fig. S11). The increase in actin anisotropy for iMSCs across the stiffness groups is consistent and highly significant, whereas for MSCs the increase between the Low to Med stiffness is greater than from Med to High stiffness (Supplementary Fig. S12). Representative quantifications of low (1.14) and high (3.26) nuclear YAP values (Fig. 3e) show brighter nuclear fluorescence for higher nuclear YAP values. The representative quantification of high (0.32) actin anisotropy (Fig. 3f) show actin fibers with prevailing directionality. Representative quantifications of low (Fig. 3g) and high (Fig. 3h) pFAK length feature the observable differences in focal adhesion maturation and number of adhesions in each cell. These findings show that iMSCs are highly homogeneous and mechanoresponsive to matrix stiffness.

### iMSCs are more homogeneous and mechanosensitive than MSCs

**Figure 4:**
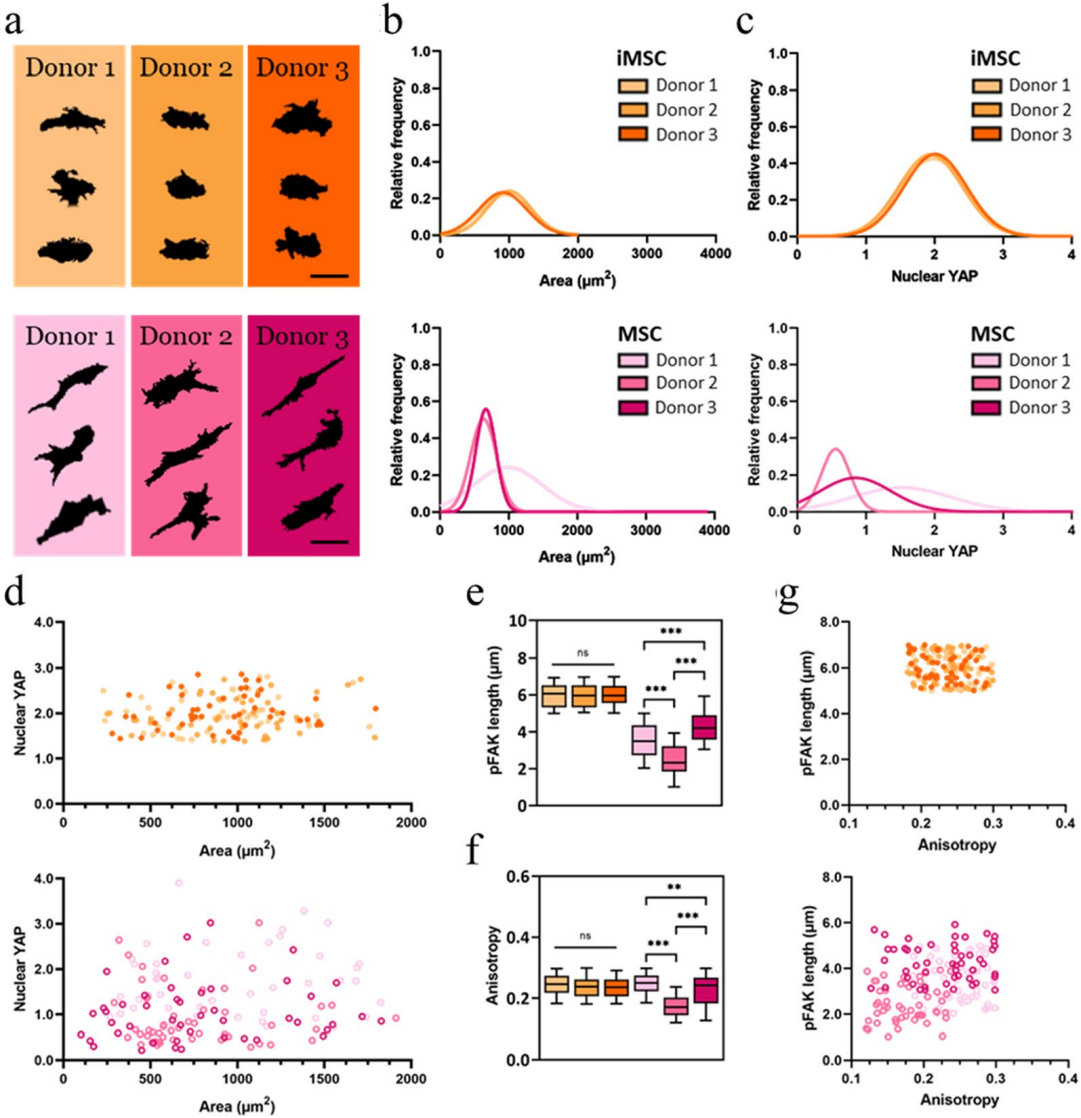
Morphological and cellular mechanosensing comparison between iMSCs and MSCs on Med stiffness hydrogels. (**a**) Representative cell silhouettes of 3 iMSC (top) and MSC (bottom) cell lines. Histograms of (**b**) cell area and (**c**) nuclear YAP of 3 iMSC (top) and MSC (bottom) cell lines. (**d**) Nuclear YAP versus area scatter plot of 3 iMSC (top) and MSC (bottom) cell lines. Whisker plots of (**e**) pFAK length and (**f**) actin anisotropy of iMSC (3 left whisker plots) and MSC (right whisker plots) cell lines. (**g**) Scatter plots of pFAK length versus actin anisotropy of 3 iMSC (top) and MSC (bottom) cell lines. Scale bars: 50 µm. Box plots show 25/50/75th percentiles, whiskers show minimum/maximum; n > 50 cells per group, *p < 0.05, **p < 0.01, ***p < 0.001, *n.s.* not significant.

Based on the findings above, we performed a direct comparison between iMSC and MSC morphology and cellular mechanosensing. On Med (~ 10 kPa) stiffness substrates, representative cell silhouettes of iMSCs (Fig. 4a, top) qualitatively show little variation in cell morphology across iMSC lines, whereas cell silhouettes of MSCs (Fig. 4a, bottom) show increased elongation and more variability in morphology across MSC lines. Cell area for iMSCs ranged from 904 ± 355 µm^2^ to 983 ± 348 µm^2^, which is a smaller range than for MSCs (758 ± 364 µm^2^ to 1039 ± 561 µm^2^) on Med stiffness hydrogels. Differences between iMSC and MSC morphology were not as pronounced on Low or High stiffness hydrogels (Supplementary Fig. S13).

Histograms of cell area show a homogeneous distribution for iMSCs (Fig. 4b, top). In contrast, although MSC lines 2 and 3 had a homogeneous cell area distribution, there was significant heterogeneity observed in MSC line 1 (Fig. 4b, bottom). Histograms of nuclear YAP show that iMSC lines peak at a nuclear YAP ratio of ~ 2 (Fig. 4c, top), whereas there is significant heterogeneity observed in nuclear YAP values across MSC lines (Fig. 4c, bottom). In the Low and High stiffness groups there was consistent homogeneity (iMSCs) and heterogeneity (MSCs) observed for cell area (Supplementary Fig. S14), circularity (Supplementary Fig. S15), aspect ratio (Supplementary Fig. S16), and nuclear YAP localization (Supplementary Fig. S17).

Next, we examined scatter plots of nuclear YAP versus area of iMSC (Fig. 4d, top) and MSC (Fig. 4d, bottom) lines cultured on Med stiffness hydrogels. Although there is a range in area for iMSC lines, the range in nuclear YAP values is lower for iMSCs than for MSCs. This observation was also seen between iMSC and MSC lines on Low and High stiffness groups (Supplementary Fig. S18). An iMSC versus MSC comparison between pFAK length on Med stiffness hydrogels shows no significant difference in pFAK length (~ 6 µm) for iMSC lines, which contrasts the heterogeneity observed across MSC lines, with values ranging from 2.47 ± 0.82 µm to 4.27 ± 0.83 µm (Fig. 4e).

Actin anisotropy values for iMSCs were also consistent across different lines while for MSCs actin anisotropy was significantly different across cell lines (Fig. 4f). These findings were consistent for pFAK length (Supplementary Fig. S19) and actin anisotropy (Supplementary Fig. S20) between iMSCs and MSCs cultured on Low and High stiffness hydrogels. Single cell scatter plots of pFAK length as a function of actin anisotropy for iMSCs and MSCs on Med stiffness hydrogels reveal tight clustering of data points for iMSC lines (Fig. 4g, top), whereas data points for MSCs were more scattered (Fig. 4g, bottom). This was also observed for iMSCs and MSCs cultured on Low and High stiffness hydrogels (Supplementary Fig. S21). Taken together, a direct comparison between MSCs and iMSCs atop hydrogels of varying stiffness shows that iMSC morphology and mechanosensitivity is significantly more consistent than for MSCs.

## Conclusions

In this study, we derive iMSCs from iPSCs and demonstrate that iMSCs are more homogeneous and mechanosensitive than MSCs isolated from adult tissues. This finding resulted from evaluating iMSC morphology and matrix mechanosensing on mechanically defined 2D hydrogels and motivates future studies that investigate iMSC-material interactions in more complex and physiologically relevant environments. To this end, the thiol-norbornene chemistry used here can be easily adapted to form hydrogels that support 3D cell culture and spatial patterning of biophysical and biochemical signals^39^. Due to their remarkable sensitivity and homogeneity, iMSCs could be a viable source for large scale manufacturing of human stem cells for both autogenic and allogeneic cell therapies. As we continue to increase our understanding of iMSC-material interactions, we also believe that iMSCs will emerge as a new class of cells for regenerative medicine and tissue engineering applications.

## Methods

### Derivation and characterization of human iMSC lines

Fibroblasts from skin biopsies were programmed into iPSCs using the Global Stem Cell Array® as previously reported^21^. All methods were carried out in accordance with relevant guidelines and regulations. All experimental protocols were approved by The New York Stem Cell Foundation Research Institute. Skin biopsies were shared as deidentified following written informed consent. Generated iPSC lines were characterized via confirmation of pluripotency markers OCT4 and TRA-1-60 using immunostaining and fluorescence imaging. Additional characterization was performed via global surface marker profiling using a BD Lyoplate Human Cell Surface Marker Screening Panel (BD Biosciences) per manufacturer’s instructions. Using chemically defined differentiation medium, iPSC lines were differentiated towards osteoblasts, chondrocytes, or adipocytes and evaluated using von Kossa (calcium deposition), Alcian blue (glycosaminoglycans, GAGs), or Oil red O (intracellular triglycerides), respectively.

### Macromer synthesis

Sodium hyaluronate (NaHA) was first converted to its tetrabutylammonium salt (HA-TBA). To synthesize HANor, the carboxylic acid residues of HA-TBA were modified with 5-norbornene-2-methylamine (~ 50% of repeat units were functionalized with Nor-). To synthesize HANorMe, the hydroxyl residues of HANor were modified with methacrylic anhydride (~ 75% of repeat units were functionalized with Me-). To biofunctionalize HANorMe with RGD, a Michael addition reaction between thiolated RGD (cRGD) peptide and methacrylates was performed (2 mM final cRGD concentration). Representative ^1^H NMR spectra used to calculate percent of HA repeat units functionalized with Nor- and Me- is in Supplementary Fig. S5.

### Hydrogel synthesis and mechanical testing

HANor was dissolved in phosphate buffer saline (PBS) at 3 wt% with varying amounts of DTT and 0.05 wt% I2959. The prepolymer solution (80 µL) was pipetted into a silicone mold (11 mm Ø, 0.5 mm h) and irradiated with UV light (10 min, 10 mW/cm^2^). Individual hydrogels were removed from the molds and placed in 1 mL of PBS to swell overnight at 37 °C before mechanical testing. Compressive moduli were determined using a Shimadzu EZ-SX Mechanical Tester running at a constant strain rate of 10%/min. The modulus was calculated from the slope of the stress–strain curve between 10 and 20% strain (n ≥ 3).

### Human MSC and iPSC cell culture

Stem cell lines from the New York Stem Cell Foundation Research Institute expanded to passage 4 in growth media [α-MEM supplemented with 10% (v/v) FBS (Gibco), 1% (v/v) penicillin–streptomycin (Invitrogen)] were used in all experiments. Human mesenchymal stem cell lines (Lonza) were expanded to passage 4 using the culture media described above. Cells were then cultured on top of Low, Medium, or High stiffness hydrogels (3000 cells/cm^2^).

### Immunofluorescence staining, imaging, and image analysis

After 3 days in culture, stem cell-laden hydrogels were fixed, permeabilized, and stained with appropriate antibodies (YAP, pFAK), phalloidin (actin), and/or Hoescht (nuclei). Samples were then imaged using a Nikon A1 confocal microscope and ImageJ software was used to calculate morphology (area, circularity, aspect ratio) and cellular mechanosensing (pFAK morphology, actin anisotropy, nuclear YAP) parameters.

### Statistical analysis

All data are from three independent biological experiments. At least 50 cells per treatment and biological experiment were quantified. For three group comparisons, one way ANOVA between groups (α = 0.05) was performed using GraphPad Prism. If the results of the ANOVA were found to be significant, post hoc analysis was performed using the Tukey multiple comparisons test to compare results among groups. Hierarchical clustering of surface marker data was generated using the analysis software Morpheus (Broad Institute) based on Euclidean distance.

## Supplementary Information

Supplementary Information.
