# Supplementary material for: Human induced mesenchymal stem cells display increased sensitivity to matrix stiffness
Source: Sci Rep. 2022 May 19;12:8483. doi: 10.1038/s41598-022-12143-2 (PMC9119934; doi:10.1038/s41598-022-12143-2)
Supplement: Supplementary file 1 — Supplementary Information. [file 41598_2022_12143_MOESM1_ESM.docx]

Supplementary Information

Human induced mesenchymal stem cells display increased sensitivity to matrix stiffness

Kirstene A. Gultian^1^, Roshni Gandhi^1^, Khushi Sarin^1^, Martina Sladkova-Faure^2^, Matthew Zimmer^2^, Giuseppe Maria de Peppo^2^, Sebastián L. Vega^1,*^

^1^Department of Biomedical Engineering, Rowan University, Glassboro, New Jersey 08028, USA

^2^The New York Stem Cell Foundation Research Institute, New York, New York 10019, USA

^*^Correspondence: vegas@rowan.edu | @theVegaLab (S.L.V.)

**This file contains:**

21 Supplementary Figures, 1 Supplementary Table, and Supplementary Methods.

**Supplementary Figures**

**
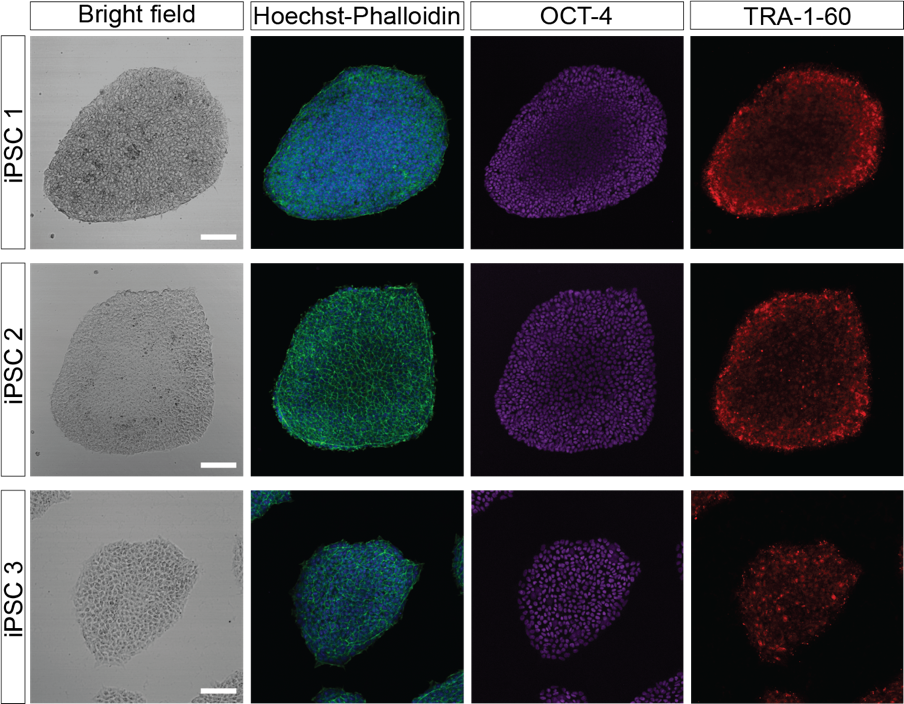
**

**Supplementary Figure S1**. **Morphology and expression of pluripotency markers**. Representative bright field images show that human iPSC lines form colonies. Representative fluorescence images show consistent colony morphology (phalloidin, green; Hoescht, blue) and that human iPSC lines are positive for pluripotency markers OCT-4 (purple) and TRA-1-60 (red). Scale bar: 100 µm.

**
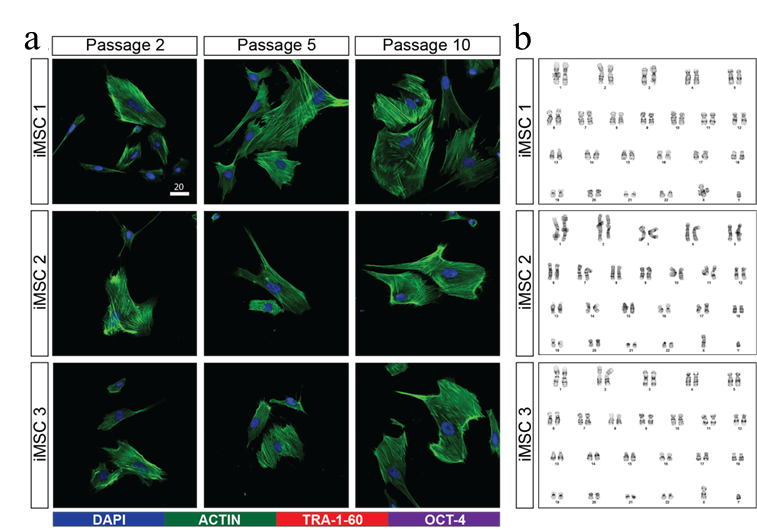
**

**Supplementary Figure S2. Lack of pluripotency and genomic integrity of human iMSCs.** (a) Representative fluorescent images show that human iMSCs lack expression of TRA-1-60 (red) and OCT-4 (purple) pluripotency markers. Scale bar, 20 µm. (b) G-banding of derived human iMSC lines.

**
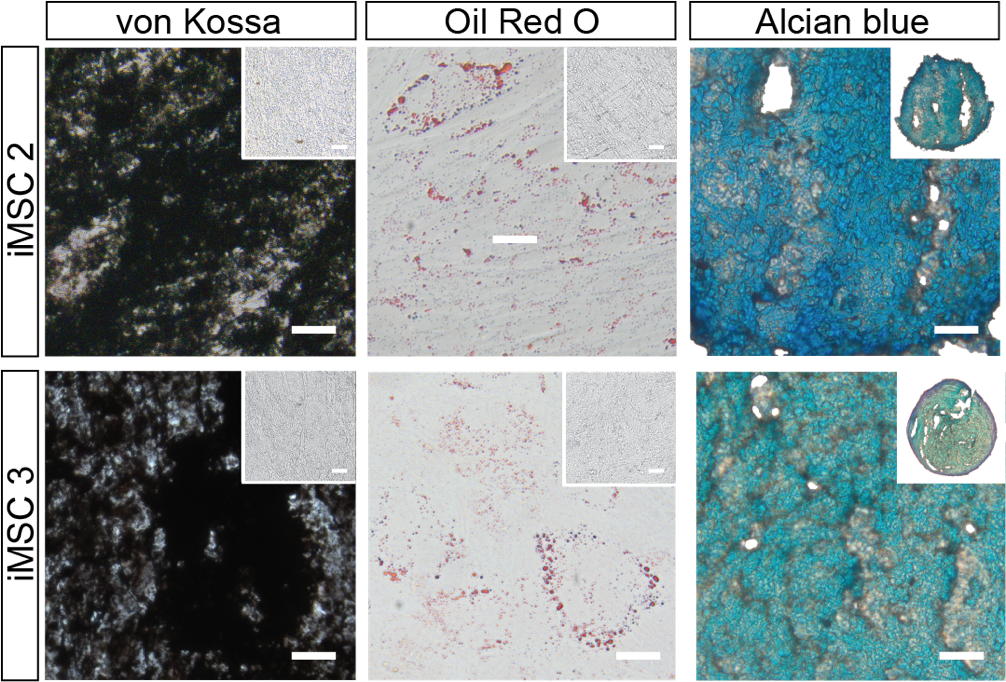
**

**Supplementary Figure S3. Multidifferentiation potential of human iMSC lines.** Micrographs showing the osteogenic (von Kossa), adipogenic (Oil Red O), and chondrogenic (Alcian blue) differentiation potential of human iMSCs (lines 2 and 3). Insets represent negative controls and full-size cartilage spheroids. Scale bars, 20 µm.

**
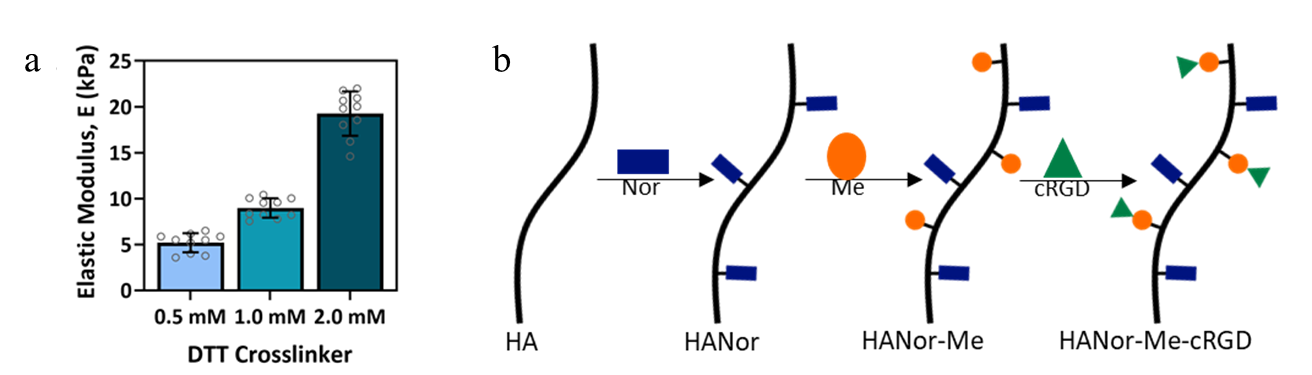
**

**Supplementary Figure S4. Hydrogel mechanics characterization and macromer design.** (a) Click crosslinking reaction between norbornenes in HA macromer and dithiothreitol (DTT) was used to form hydrogels with a range in mechanics by varying crosslinker concentrations (Low, 0.5 mM; Med, 1.0 mM, High, 2.0 mM). (b) Scheme for forming HANor-Me-cRGD macromers from HA. Bar graphs represent the mean and error bars represent standard deviation.

**
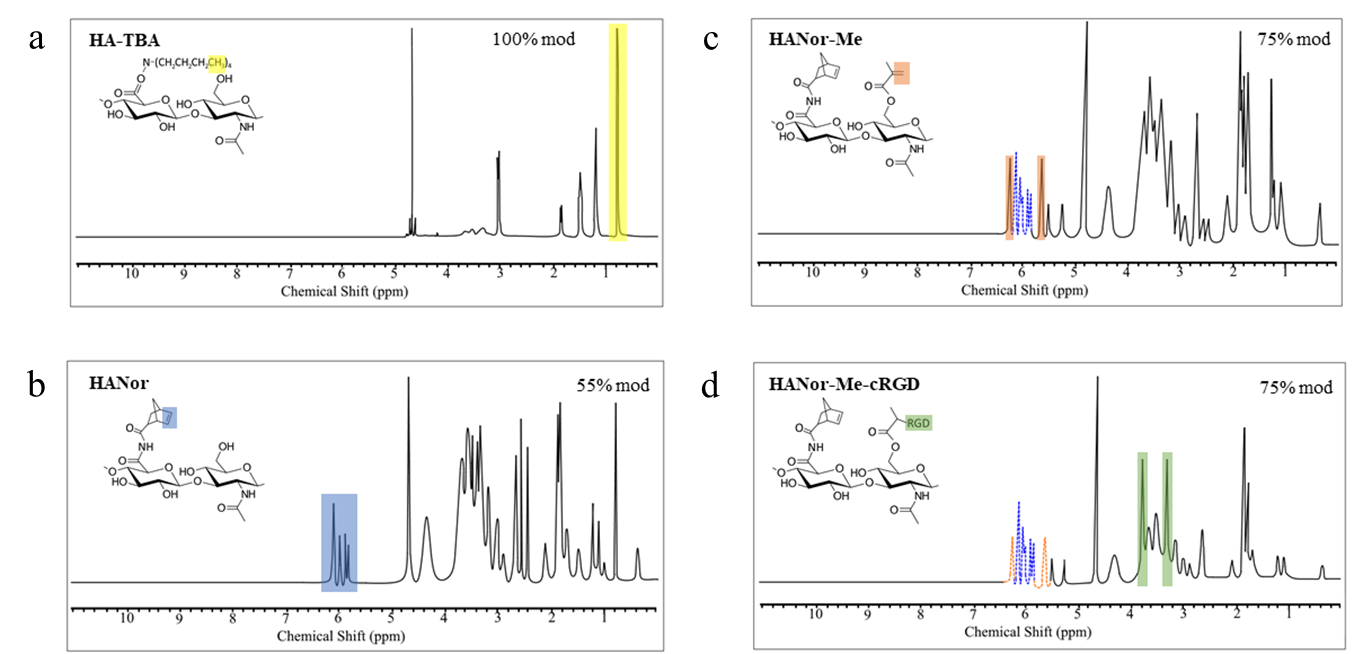
**

**Supplementary Figure S5. ^1^H NMR spectra of hyaluronic acid with coupled functionalities**. ^1^H NMR of (a) tetrabutylammonium salt of hyaluronic acid (HA-TBA) shows 100% modification with TBA (yellow peak), (b) HA coupled with norbornene (HANor) shows 55% modification with Nor- (blue peaks), (c) HANor coupled with methacrylates (HANorMe) shows 75% modification with Me- (orange peaks), and (d) HANorMe coupled with thiolated RGD peptide (HANorMe-cRGD) shows 75% modification with cRGD peptide (green peaks).


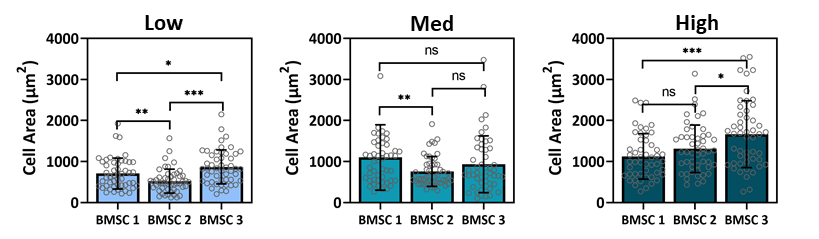


**Supplementary Figure S6. Cell spread area for MSCs cultured atop Low, Med, and High stiffness hydrogels**. n > 50 cells per group, bar graphs represent the mean and error bard represent standard deviation, *p < 0.1, **p < 0.01, ***p < 0.001, ns = no significant difference.

**
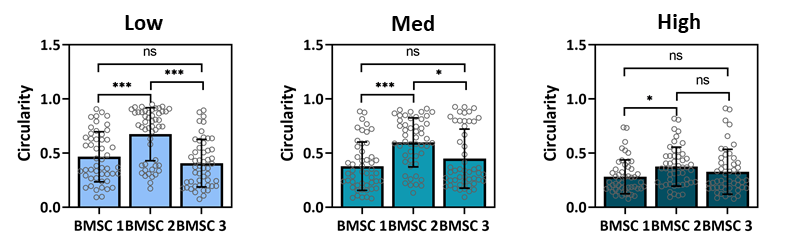
**

**Supplementary Figure S7. Cell circularity for MSCs cultured atop Low, Med, and High stiffness hydrogels**. n > 50 cells per group, bar graphs represent the mean and error bard represent standard deviation, *p < 0.1, **p < 0.01, ***p < 0.001, ns = no significant difference.

**
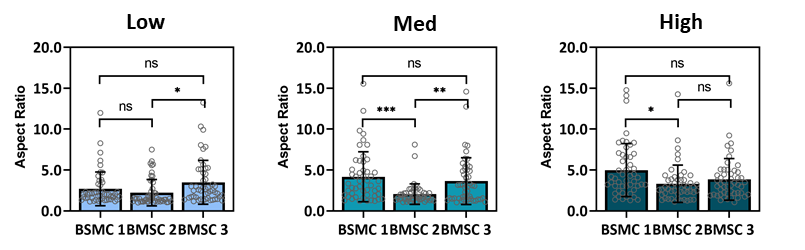
**

**Supplementary Figure S8. Cell aspect ratio for MSCs cultured atop Low, Med, and High stiffness hydrogels**. n > 50 cells per group, bar graphs represent the mean and error bard represent standard deviation, *p < 0.1, **p < 0.01, ***p < 0.001, ns = no significant difference.

**
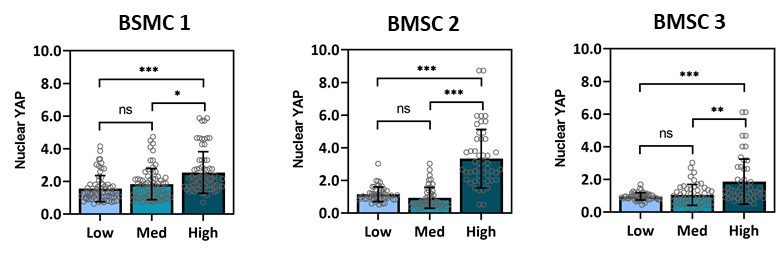
**

**Supplementary Figure S9. YAP nuclear localization for MSCs cultured atop Low, Med, and High stiffness hydrogels**. n > 50 cells per group, bar graphs represent the mean and error bard represent standard deviation, *p < 0.1, **p < 0.01, ***p < 0.001, ns = no significant difference.

**
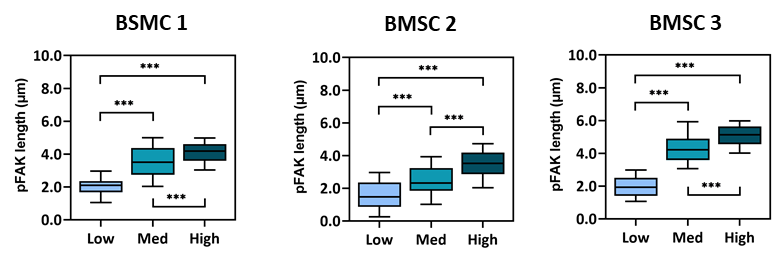
**

**Supplementary Figure S10. Phosphorylated focal adhesion kinase (pFAK) length for MSCs cultured atop Low, Med, and High stiffness hydrogels**. n > 50 cells per group, box plots show 25/50/75th percentiles, whiskers show minimum/maximum, *p < 0.1, **p < 0.01, ***p < 0.001, ns = no significant difference.

**
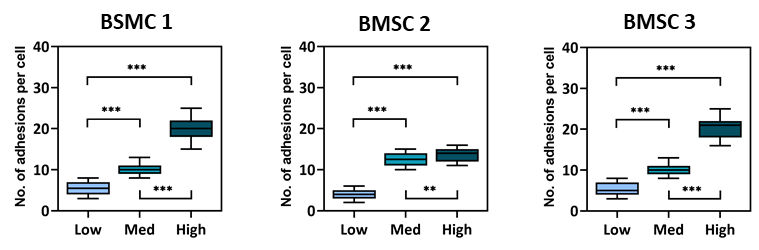
**

**Supplementary Figure S11. Number of pFAK adhesions per cell for MSCs cultured atop Low, Med, and High stiffness hydrogels**. n > 50 cells per group, box plots show 25/50/75th percentiles, whiskers show minimum/maximum, *p < 0.1, **p < 0.01, ***p < 0.001, ns = no significant difference.

**
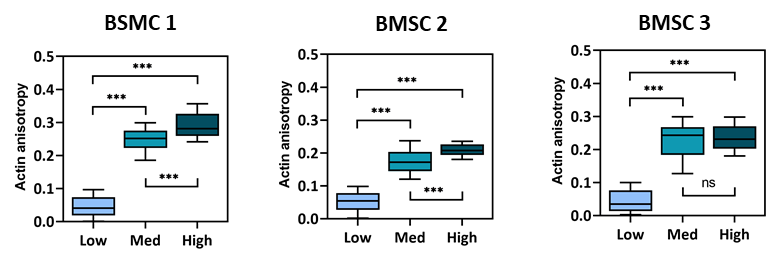
**

**Supplementary Figure S12. Actin fiber anisotropy for MSCs cultured atop Low, Med, and High stiffness hydrogels**. n > 50 cells per group, box plots show 25/50/75th percentiles, whiskers show minimum/maximum, *p < 0.1, **p < 0.01, ***p < 0.001, ns = no significant difference.


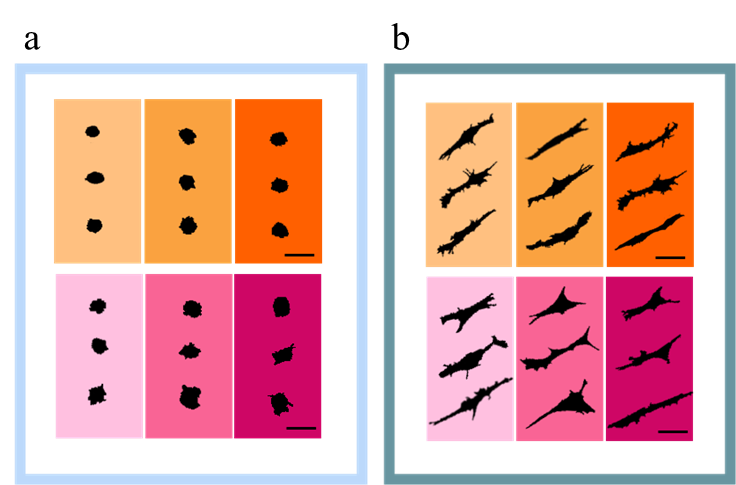


**Supplementary Figure S13. Representative silhouettes for all iMSC and MSC lines atop Low and High stiffness hydrogels.** (a) Top: iMSC lines 1 to 3 (left to right), Bottom: BMSC lines 1 to 3 (left to right) on Low hydrogels. (b) Top: iMSC lines 1, 2, and 3 (left to right), Bottom: MSC lines 1, 2, and 3 (left to right) on High hydrogels. Scale bars: 50 µm.


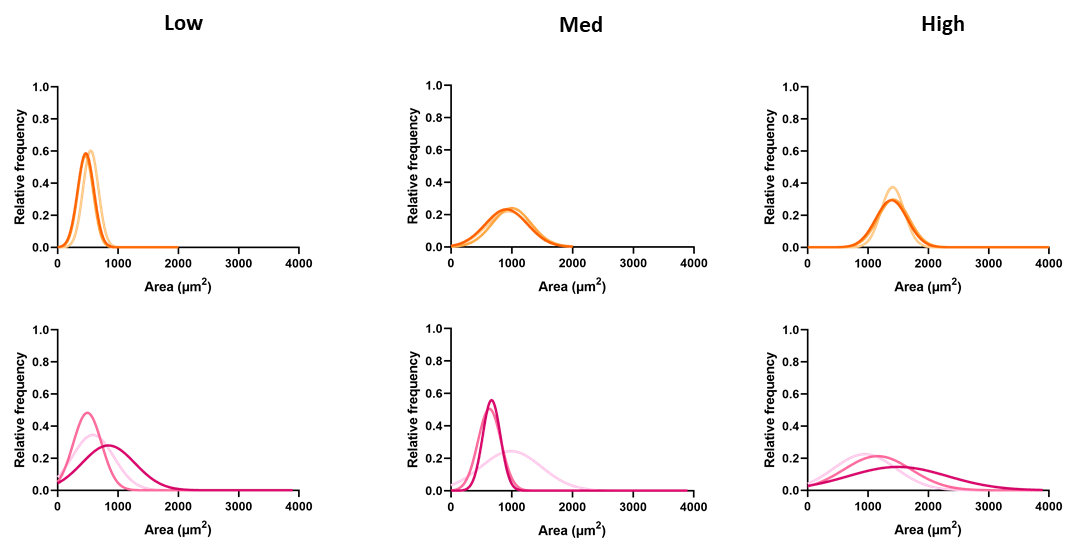


**Supplementary Figure S14. Frequency distribution of spread area for all iMSC and MSC lines atop Low, Med, and High stiffness hydrogels.** Top: iMSC lines 1, 2, and 3, Bottom: MSC lines 1, 2, and 3.

**
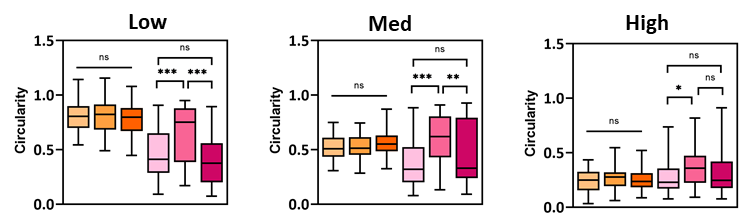
**

**Supplementary Figure S15. Circularity for iMSC (boxes 1 to 3) and MSC (boxes 4 to 6) lines atop Low, Med, and High stiffness hydrogels.** n > 50 cells per group, box plots show 25/50/75th percentiles, whiskers show minimum/maximum, *p < 0.1, **p < 0.01, ***p < 0.001, ns = no significant difference.

**
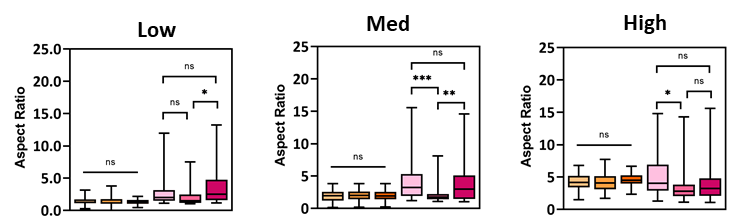
**

**Supplementary Figure S16. Aspect ratio for iMSC (boxes 1 to 3) and MSC (boxes 4 to 6) lines atop Low, Med, and High stiffness hydrogels.** n > 50 cells per group, box plots show 25/50/75th percentiles, whiskers show minimum/maximum, *p < 0.1, **p < 0.01, ***p < 0.001, ns = no significant difference.

**
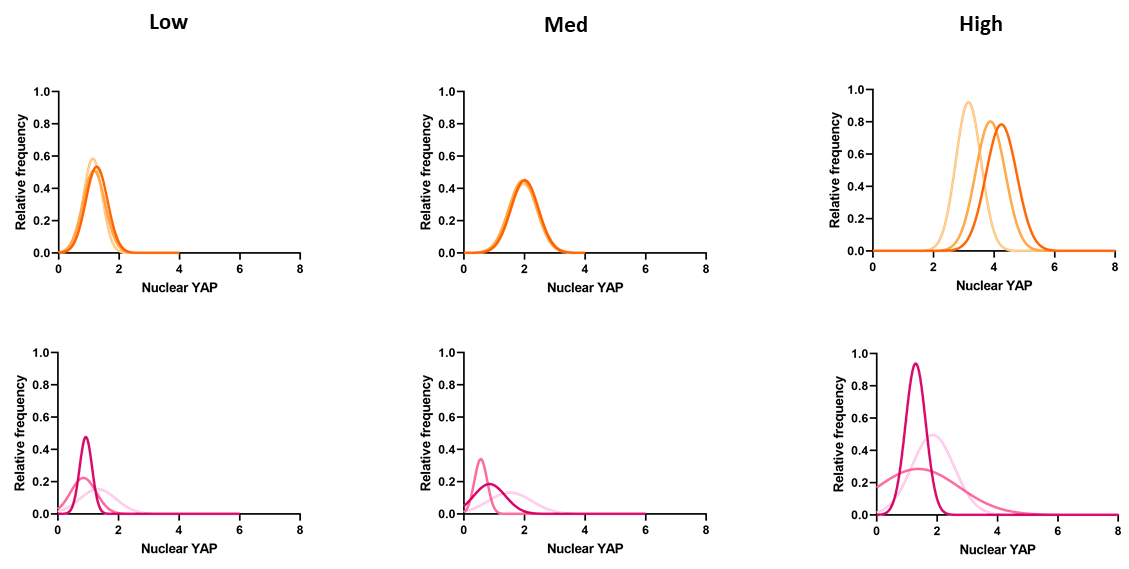
**

**Supplementary Figure S17. Frequency distribution of nuclear YAP for all iMSC and MSC lines atop Low, Med, and High stiffness hydrogels.** Top: iMSC lines 1, 2, and 3, Bottom: MSC lines 1, 2, and 3.

**
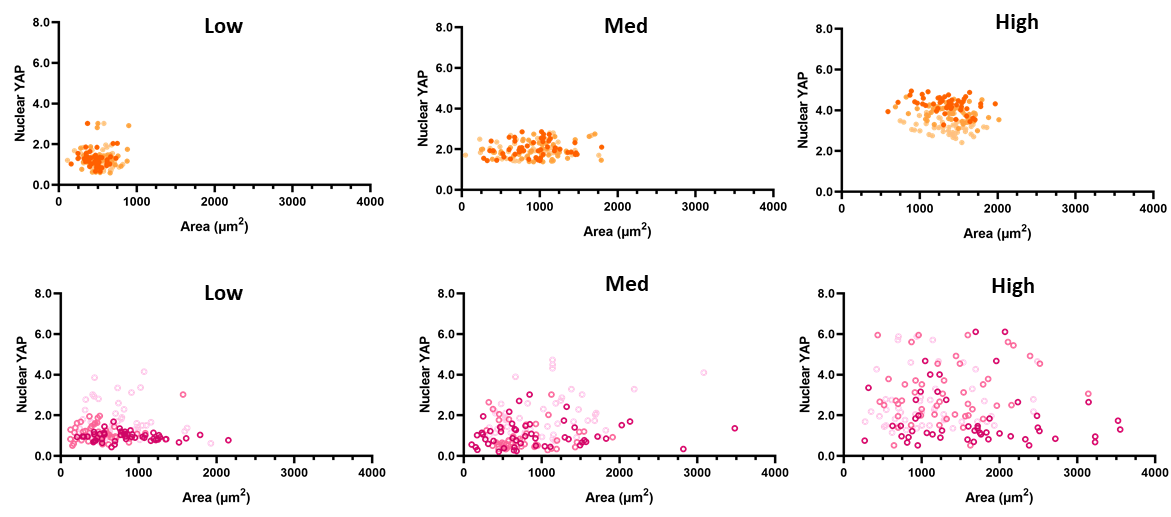
**

**Supplementary Figure S18. Scatter dot plot of nuclear YAP as a function of cell area for all iMSC and MSC lines atop Low, Med, and High stiffness hydrogels.** Top: iMSC lines 1, 2, and 3, Bottom: MSC lines 1, 2, and 3.

**
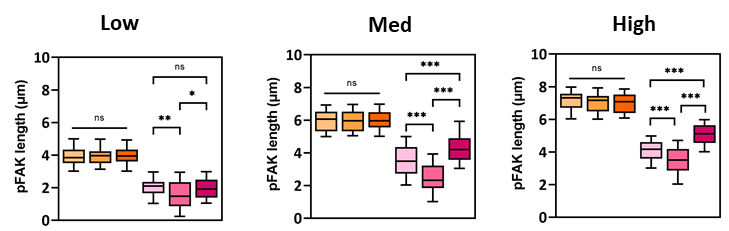
**

**Supplementary Figure S19. Phosphorylated focal adhesion kinase (pFAK) length for iMSC (boxes 1 to 3) and MSC (boxes 4 to 6) lines atop Low, Med, and High stiffness hydrogels.** n > 50 cells per group, box plots show 25/50/75th percentiles, whiskers show minimum/maximum, *p < 0.1, **p < 0.01, ***p < 0.001, ns = no significant difference.

**
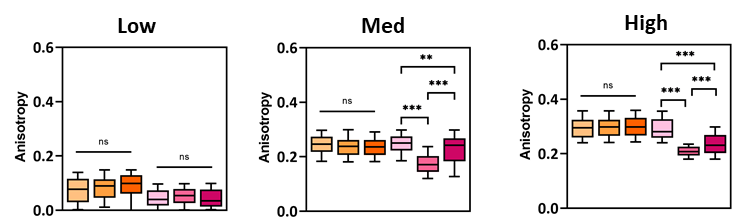
**

**Supplementary Figure S20. Actin anisotropy for iMSC (boxes 1 to 3) and MSC (boxes 4 to 6) lines atop Low, Med, and High stiffness hydrogels.** n > 50 cells per group, box plots show 25/50/75th percentiles, whiskers show minimum/maximum, *p < 0.1, **p < 0.01, ***p < 0.001, ns = no significant difference.

**
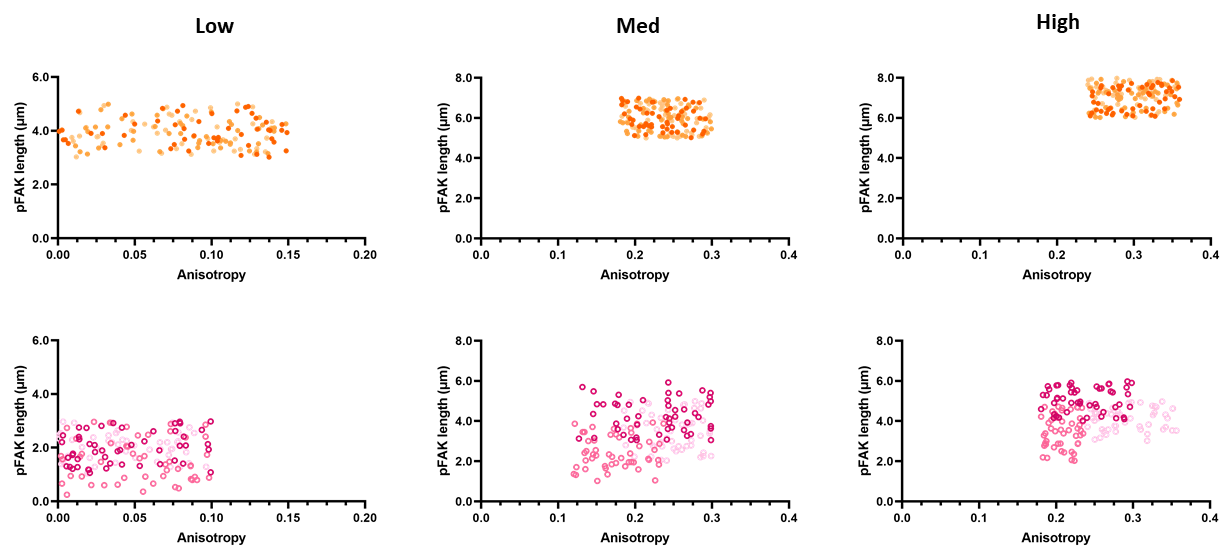
**

**Supplementary Figure S21. Scatter dot plot of pFAK length as a function of actin anisotropy for all iMSC and MSC lines on Low, Med, and High stiffness hydrogels.** Top: iMSC lines 1, 2, and 3, Bottom: MSC lines 1, 2, and 3.

**Supplementary Table**

**Supplementary Table S1. Global surface marker profile.** Percentage of positive human iPSCs at passage 2 (P2), 5 (P5), 10 (P10), and MSCs at P5 for 246 surface markers.

| **Surface marker** | **iMSC 1**  **P2** | **iMSC 2**  **P2** | **iMSC 3**  **P2** | **iMSC 1**  **P5** | **iMSC 2**  **P5** | **iMSC 3**  **P5** | **iMSC 1**  **P10** | **iMSC 2**  **P10** | **iMSC 3**  **P10** | **BMSC**  **P5** |
| --- | --- | --- | --- | --- | --- | --- | --- | --- | --- | --- |
| **CD1a** | 0.715 | 0.841 | 0.468 | 0.459 | 0.498 | 0.591 | 0.904 | 0.273 | 0.694 | 0.188 |
| **CD1b** | 0.555 | 0.314 | 0.702 | 0.615 | 0.363 | 0.603 | 0.566 | 0.188 | 0.84 | 0.327 |
| **CD1d** | 0.451 | 0.303 | 0.48 | 0.551 | 0.276 | 0.693 | 0.978 | 0.304 | 0.398 | 0.278 |
| **CD2** | 0.972 | 0.802 | 0.681 | 0.276 | 0.436 | 0.557 | 0.386 | 0.0552 | 0 | 0.195 |
| **CD3** | 0.84 | 0.4 | 0.874 | 0.334 | 1.12 | 0.498 | 3.48 | 1.3 | 2.79 | 0.435 |
| **CD4** | 1.08 | 0.709 | 0.838 | 0.581 | 0.18 | 0.378 | 0.41 | 0.353 | 0.4 | 2.61 |
| **CD4v4** | 0.862 | 0.766 | 0.845 | 0.571 | 0.246 | 0.621 | 0.345 | 0.264 | 0.82 | 2.01 |
| **CD5** | 0.714 | 0.549 | 0.903 | 0.477 | 0.202 | 0.412 | 0.582 | 0.17 | 0.177 | 0.617 |
| **CD6** | 0.815 | 0.386 | 0.476 | 0.635 | 0.268 | 0.421 | 0.38 | 0.0709 | 1.18 | 0.255 |
| **CD7** | 2.63 | 0 | 0 | 0.805 | 0.497 | 0.34 | 1.22 | 0.436 | 0.208 | 0.426 |
| **CD8a** | 1.19 | 0.651 | 1.12 | 0.497 | 0.361 | 0.596 | 0.763 | 0.0648 | 0.629 | 0.278 |
| **CD8b** | 0.616 | 0.244 | 0.615 | 0.573 | 0.435 | 0.636 | 0.418 | 0.0725 | 1.69 | 0.52 |
| **CD10** | 70.2 | 17.2 | 85.3 | 40.9 | 7.72 | 67.7 | 44.7 | 11.8 | 47.9 | 11.7 |
| **CD11a** | 0.929 | 0.37 | 0.753 | 0.803 | 0.264 | 0.124 | 0.409 | 0.124 | 1.68 | 0.206 |
| **CD11b** | 0.712 | 0.58 | 0.856 | 0.545 | 0.259 | 0.345 | 0.702 | 0.403 | 0.546 | 0.274 |
| **CD11c** | 0.62 | 0.695 | 0.513 | 0.575 | 0.349 | 0.508 | 0.567 | 0.177 | 1.16 | 0.442 |
| **CD13** | 96.7 | 90.7 | 97.8 | 80.8 | 64.8 | 69 | 78.3 | 68 | 62.6 | 91.6 |
| **CD14** | 0.757 | 0.938 | 0.879 | 0.423 | 0.414 | 0.474 | 1.22 | 0.397 | 0.605 | 0.326 |
| **CD15** | 0.806 | 0.62 | 0.655 | 1.13 | 0.369 | 1.42 | 0.822 | 0.466 | 6.44 | 0.355 |
| **CD15s** | 0.63 | 0.773 | 0.846 | 0.682 | 0.397 | 0.631 | 1.02 | 0.233 | 0.525 | 0.753 |
| **CD18** | 1.12 | 0.724 | 0.663 | 0.647 | 0.281 | 0.582 | 0.692 | 0.623 | 0.977 | 0.776 |
| **CD19** | 0.445 | 0.358 | 0.782 | 0.586 | 0.242 | 0.438 | 0.41 | 0.215 | 0.234 | 0.412 |
| **CD20** | 0.569 | 0.224 | 0.634 | 0.367 | 0.211 | 0.394 | 0.483 | 0 | 0 | 0.296 |
| **CD21** | 0.92 | 0.365 | 0.306 | 0.459 | 0.189 | 0.46 | 0.587 | 0 | 0.791 | 0.285 |
| **CD22** | 0.668 | 0.726 | 0.74 | 0.895 | 0.206 | 0.677 | 1.29 | 0.254 | 2.54 | 0.738 |
| **CD23** | 0.531 | 0.446 | 0.549 | 0.456 | 0.188 | 0.336 | 0.949 | 0.177 | 0.956 | 0.237 |
| **CD24** | 14 | 50 | 3.96 | 12.1 | 87.1 | 1.45 | 2.02 | 18 | 1.06 | 3.66 |
| **CD25** | 0.784 | 0.779 | 0.72 | 0.586 | 0.402 | 0.396 | 0.272 | 0.116 | 0.581 | 0.363 |
| **CD26** | 21.9 | 11.6 | 6.58 | 15.4 | 2.21 | 3.21 | 14.8 | 2.06 | 3.31 | 6.9 |
| **CD27** | 0.489 | 0.828 | 0.392 | 0.53 | 0.32 | 0.366 | 0.55 | 0.34 | 0.988 | 0.274 |
| **CD28** | 0.652 | 0.67 | 0.466 | 0.484 | 0.319 | 0.327 | 0.792 | 0.0575 | 0.573 | 0.324 |
| **CD29** | 97.9 | 94.4 | 94.2 | 83.7 | 47.2 | 74.8 | 75.2 | 57.3 | 56.3 | 86.5 |
| **CD30** | 0.505 | 0.798 | 0.421 | 0.476 | 0.434 | 0.563 | 0.435 | 0.18 | 1.6 | 0.545 |
| **CD31** | 0.558 | 0.521 | 0.331 | 0.74 | 0.244 | 0.745 | 1.14 | 0.348 | 1.08 | 0.468 |
| **CD32** | 0.68 | 1.19 | 0.566 | 0.573 | 0.217 | 0.646 | 0.859 | 0.266 | 0.726 | 0.759 |
| **CD33** | 0.562 | 0.435 | 0.595 | 0.483 | 0.215 | 0.346 | 0.442 | 0.0603 | 0.401 | 0.868 |
| **CD34** | 1.17 | 0.566 | 2.22 | 1.21 | 0.292 | 1.31 | 0.637 | 0.203 | 0.63 | 0.904 |
| **CD35** | 0.591 | 0.792 | 0.33 | 0.447 | 0.225 | 0.544 | 0.216 | 0.158 | 0.261 | 0.364 |
| **CD36** | 1.02 | 0.759 | 0.714 | 0.718 | 0.2 | 0.575 | 0.913 | 0.171 | 0.899 | 0.903 |
| **CD37** | 0.826 | 3.4 | 0.936 | 0.442 | 0.188 | 0.327 | 0.147 | 0 | 0.211 | 0.446 |
| **CD38** | 2.83 | 1.52 | 1 | 1.03 | 0.105 | 0.674 | 0.292 | 0 | 0.194 | 0.416 |
| **CD39** | 0.917 | 0.594 | 1.37 | 0.706 | 0.401 | 0.757 | 0.601 | 0.361 | 0.671 | 0.631 |
| **CD40** | 9.11 | 4.44 | 12.5 | 7.99 | 2.2 | 8.3 | 1.31 | 1.03 | 8.09 | 0.481 |
| **CD41a** | 0.475 | 0.803 | 0.502 | 0.379 | 0.453 | 0.358 | 0.686 | 0.0586 | 1.09 | 0.492 |
| **CD41b** | 0.895 | 1.04 | 0.81 | 0.517 | 0.455 | 0.574 | 0.901 | 0.187 | 0.956 | 0.405 |
| **CD42a** | 0.874 | 0.625 | 0.55 | 0.804 | 0.325 | 0.472 | 0.371 | 0.271 | 0.924 | 0.293 |
| **CD42b** | 0.487 | 0.771 | 0.345 | 0.475 | 0.245 | 0.448 | 0.637 | 0.165 | 0.347 | 0.574 |
| **CD43** | 1.16 | 1.07 | 0.746 | 0.391 | 0.247 | 0.56 | 0.278 | 0 | 0.397 | 0.525 |
| **CD44** | 100 | 90.8 | 90.2 | 95.7 | 98.1 | 87 | 90.9 | 89.2 | 91.8 | 98.5 |
| **CD45** | 0.972 | 0.695 | 0.519 | 0.467 | 0.364 | 0.204 | 0.681 | 0.191 | 0.634 | 0.29 |
| **CD45RA** | 0.934 | 0.611 | 0.721 | 0.526 | 0.526 | 0.698 | 0.726 | 0.18 | 1.08 | 0.351 |
| **CD45RB** | 0.615 | 0.537 | 0.466 | 0.4 | 0.352 | 0.567 | 0.284 | 0.235 | 1.2 | 0.53 |
| **CD45RO** | 0.792 | 0.915 | 0.927 | 0.355 | 0.3 | 0.461 | 0.785 | 0.235 | 0 | 0.296 |
| **CD46** | 98.8 | 99.2 | 98.9 | 96.8 | 88.5 | 95.6 | 91.1 | 79.5 | 88.8 | 94.1 |
| **CD47** | 98.1 | 89.6 | 95.6 | 91.7 | 42 | 82.6 | 68.8 | 46.1 | 67.2 | 89.8 |
| **CD48** | 0.559 | 0.639 | 0.547 | 0.546 | 0.455 | 0.516 | 0.625 | 0.181 | 0.203 | 0.343 |
| **CD49a** | 42.3 | 50.3 | 49.9 | 12 | 11.1 | 32.4 | 1.54 | 9.64 | 13.1 | 44.3 |
| **CD49b** | 89.8 | 59 | 84.3 | 94 | 91.9 | 90.4 | 98.1 | 92.4 | 97.3 | 89.5 |
| **CD49c** | 99.3 | 93.8 | 98 | 83.7 | 95.2 | 76.2 | 91.9 | 81.3 | 82.6 | 76.1 |
| **CD49d** | 56.1 | 68.9 | 67.9 | 78.4 | 78.2 | 69.2 | 48.5 | 66.7 | 11.4 | 60.3 |
| **CD49e** | 91.8 | 86.1 | 90.3 | 91.2 | 97.2 | 89.5 | 91.6 | 96.6 | 93 | 90.1 |
| **CD50** | 2.58 | 1.34 | 3.76 | 1.58 | 0.357 | 1.29 | 0.423 | 0.0576 | 0.409 | 6.23 |
| **CD51/61** | 50.9 | 75.6 | 18.4 | 77.3 | 62.5 | 34.9 | 73 | 66.9 | 62.4 | 50.9 |
| **CD53** | 0.212 | 0.515 | 0.474 | 0.479 | 0.172 | 0.353 | 0.588 | 0.229 | 1.31 | 0.236 |
| **CD54** | 76.7 | 47 | 57.9 | 58.7 | 62.6 | 63.4 | 77.8 | 79.9 | 82.4 | 16.9 |
| **CD55** | 74.1 | 83.1 | 89.3 | 72 | 93.7 | 90.3 | 92.2 | 94.9 | 92.3 | 98 |
| **CD56** | 5.25 | 32.8 | 1.08 | 1.48 | 4 | 0.498 | 0.724 | 0.644 | 0.828 | 6.1 |
| **CD57** | 29.3 | 35.7 | 24.2 | 47.4 | 26.3 | 27.4 | 73.8 | 41.6 | 51.4 | 68.2 |
| **CD58** | 97.7 | 90 | 95.7 | 72.9 | 91.9 | 60.9 | 83.1 | 87.8 | 88.8 | 53.3 |
| **CD59** | • | 100 | 66.7 | 99.4 | 100 | 99.2 | 100 | 100 | 60 | 98.2 |
| **CD61** | 43.7 | 71.2 | 13.9 | 59.8 | 49.8 | 19.2 | 66.9 | 56.9 | 53.9 | 25.9 |
| **CD62E** | 0.483 | 0.478 | 0.643 | 0.543 | 0.272 | 0.392 | 0.556 | 0.23 | 0.803 | 0.381 |
| **CD62L** | 0.787 | 0.726 | 0.593 | 0.504 | 0.257 | 0.547 | 0.312 | 0 | 0.391 | 0.328 |
| **CD62P** | 0.907 | 1.02 | 0.663 | 0.504 | 0.321 | 0.461 | 0.902 | 0.25 | 0.596 | 0.316 |
| **CD63** | 92.8 | 82.1 | 92.8 | 70.4 | 83.4 | 73.3 | 71.3 | 93 | 77.8 | 74 |
| **CD64** | 0.676 | 0.63 | 0.758 | 0.39 | 0.302 | 0.479 | 0.451 | 0.128 | 0.577 | 0.466 |
| **CD66(a.c.d.e)** | 7.13 | 1.64 | 4.3 | 1.13 | 0.561 | 5.97 | 0.286 | 0.38 | 1.28 | 0.592 |
| **CD66b** | 0.555 | 0.808 | 0.779 | 0.404 | 0.404 | 0.477 | 0.602 | 0.379 | 0.982 | 0.337 |
| **CD66f** | 0.792 | 0.947 | 0.929 | 0.472 | 0.323 | 0.449 | 0.275 | 0.11 | 0.409 | 0.395 |
| **CD69** | 0.331 | 0.257 | 0.726 | 0.39 | 0.302 | 0.598 | 0.414 | 0 | 0.377 | 0.194 |
| **CD70** | 6.98 | 5 | 6.52 | 2.1 | 1.62 | 2.79 | 0.594 | 3.68 | 31 | 1.78 |
| **CD71** | 90 | 84.8 | 96.5 | 98.4 | 99.4 | 95.5 | 88.9 | 93.8 | 93.2 | 92.4 |
| **CD72** | 0.909 | 0.86 | 0.833 | 0.572 | 0.438 | 0.636 | 1.56 | 0.245 | 0.621 | 0.529 |
| **CD73** | 98.8 | 96.5 | 100 | 84.9 | 99 | 80.4 | 100 | 96.1 | 97.3 | 97.5 |
| **CD74** | 0.472 | 0.768 | 0.523 | 0.813 | 0.241 | 0.565 | 0.958 | 0.475 | 0.223 | 1.62 |
| **CD75** | 0.18 | 0.579 | 0.591 | 0.735 | 0.159 | 0.574 | 0.611 | 0.255 | 0.607 | 0.365 |
| **CD77** | 0.375 | 0.524 | 0.712 | 0.788 | 0.926 | 0.757 | 0.868 | 0.503 | 1.13 | 22.1 |
| **CD79b** | 0.866 | 1.43 | 0.641 | 0.61 | 0.357 | 0.397 | 1.26 | 0.351 | 0.586 | 0.347 |
| **CD80** | 0.847 | 0.589 | 0.6 | 0.518 | 0.544 | 0.78 | 0.529 | 0.303 | 1.18 | 0.628 |
| **CD81** | 100 | 94.9 | 99.1 | 99.6 | 100 | 99.9 | 99.4 | 99.4 | 95.2 | 97 |
| **CD83** | 0.989 | 0.771 | 0.615 | 0.462 | 0.669 | 0.622 | 1.62 | 0.357 | 1.43 | 0.381 |
| **CD84** | 0.483 | 0.608 | 0.885 | 0.509 | 0.597 | 0.439 | 0.798 | 0.363 | 1.28 | 0.458 |
| **CD85** | 0.757 | 0.835 | 0.83 | 0.256 | 0.469 | 0.725 | 1.05 | 0.49 | 0.395 | 0.403 |
| **CD86** | 1.22 | 0.456 | 0.544 | 0.463 | 0.444 | 0.284 | 0.287 | 0.444 | 1.13 | 0.285 |
| **CD87** | 2.14 | 1.22 | 1.46 | 0.674 | 0.572 | 0.625 | 2.31 | 0.307 | 1.12 | 0.869 |
| **CD88** | 1.13 | 0.786 | 1.09 | 0.505 | 0.4 | 0.685 | 1.1 | 0.218 | 0.415 | 0.56 |
| **CD89** | 0.938 | 0.447 | 0.968 | 0.551 | 0.455 | 0.497 | 0 | 0.188 | 0.363 | 0.521 |
| **CD90** | 33.1 | 59.6 | 32.1 | 26 | 49 | 47.3 | 52.2 | 13.5 | 69 | 83.8 |
| **CD91** | 87.2 | 77.8 | 90.3 | 72.5 | 80.9 | 62.9 | 51.8 | 73.5 | 48.7 | 65.5 |
| **CDw93** | 0.786 | 0.461 | 1.1 | 0.766 | 0.222 | 0.444 | 0.734 | 0.0494 | 0.372 | 0.577 |
| **CD94** | 0.668 | 0.357 | 0.776 | 0.578 | 0.412 | 0.596 | 0.541 | 0.0494 | 0.725 | 0.335 |
| **CD95** | 50.9 | 52 | 46.6 | 37 | 28 | 31.7 | 51.7 | 18.7 | 29.3 | 85.7 |
| **CD97** | 2.66 | 1.28 | 4.85 | 11.3 | 4.74 | 28.4 | 4.17 | 5.95 | 12.2 | 22.1 |
| **CD98** | 99.1 | 97.2 | 98.1 | 77 | 91.7 | 76.9 | 87.9 | 83.7 | 85.8 | 74.3 |
| **CD99** | 56 | 39.2 | 53 | 42.4 | 93 | 23.3 | 16.4 | 79 | 27.8 | 47.6 |
| **CD99R** | 1.17 | 0.962 | 1.23 | 1.82 | 36.3 | 0.608 | 0.358 | 12.7 | 1.64 | 1.88 |
| **CD100** | 1.35 | 1.06 | 1.08 | 0.334 | 0.302 | 0.322 | 0.513 | 0.161 | 0 | 0.237 |
| **CD102** | 1.47 | 0.743 | 1.94 | 0.708 | 0.411 | 1.2 | 0.889 | 0.278 | 0.632 | 5 |
| **CD103** | 1.1 | 0.962 | 0.91 | 0.366 | 0.153 | 0.455 | 0.436 | 0.111 | 0.409 | 0.576 |
| **CD105** | 97.3 | 91.2 | 99 | 67.7 | 97.5 | 55.7 | 95.1 | 91.2 | 94.6 | 92 |
| **CD106** | 26.7 | 4.96 | 5.75 | 5.49 | 9.89 | 0.929 | 2.09 | 15.5 | 5.97 | 1.44 |
| **CD107a** | 17.2 | 21.2 | 21.1 | 1.9 | 8.12 | 4.43 | 6.64 | 28.1 | 9.23 | 0.878 |
| **CD107b** | 5.44 | 6.09 | 5.95 | 0.999 | 3.21 | 2.8 | 3.71 | 10.9 | 4.59 | 0.488 |
| **CD108** | 58.4 | 86.4 | 83.7 | 67.2 | 78 | 29.8 | 81.8 | 63.4 | 69.7 | 70.7 |
| **CD109** | 1.3 | 0.479 | 1.28 | 0.371 | 0.698 | 0.524 | 0.881 | 0.475 | 1.19 | 0.378 |
| **CD112** | 2.91 | 11 | 14.9 | 1.18 | 2.03 | 0.928 | 0.622 | 1.68 | 1.26 | 0.198 |
| **CD114** | 0.708 | 0.93 | 0.906 | 0.603 | 0.278 | 0.289 | 0.673 | 0.297 | 0.694 | 0.745 |
| **CD116** | 53.4 | 13.4 | 44.6 | 26.5 | 8.06 | 30.7 | 24.6 | 7.9 | 28.4 | 20.6 |
| **CD117** | 0.913 | 0.908 | 1.04 | 0.331 | 0.466 | 0.24 | 0.278 | 0.214 | 0.187 | 0.533 |
| **CD118** | 1.05 | 1.02 | 0.709 | 0.483 | 0.194 | 0.519 | 0.407 | 0.157 | 0.537 | 0.59 |
| **CD119** | 6.5 | 3.72 | 7.41 | 2.74 | 3.43 | 2.86 | 1.71 | 5.64 | 6.03 | 0.95 |
| **CD120a** | 2.07 | 1.36 | 1.69 | 0.905 | 0.319 | 1.13 | 2.21 | 1.8 | 4.32 | 1.35 |
| **CD121a** | 1.07 | 0.849 | 0.62 | 0.445 | 0.212 | 0.258 | 0.374 | 0.161 | 0.187 | 0.61 |
| **CD121b** | 0.483 | 0.535 | 0.667 | 0.697 | 0.299 | 0.348 | 0.289 | 0.17 | 0.634 | 0.405 |
| **CD122** | 1.03 | 0.536 | 0.841 | 0.485 | 0.215 | 0.203 | 0.522 | 0.0528 | 0.552 | 0.41 |
| **CD123** | 0.798 | 0.656 | 0.834 | 0.879 | 38.8 | 0.443 | 49.1 | 30.6 | 40.7 | 0.486 |
| **CD124** | 0.573 | 0.297 | 0.287 | 0.37 | 0.567 | 0.412 | 0.136 | 0.225 | 0.758 | 0.29 |
| **CD126** | 0.558 | 0.424 | 0.327 | 0.542 | 0.41 | 0.413 | 1.52 | 0.272 | 1.06 | 0.323 |
| **CD127** | 1.48 | 1.13 | 1.14 | 0.412 | 0.342 | 0.385 | 0.492 | 0.0981 | 0.337 | 0.381 |
| **CD128b** | 0.906 | 0.354 | 0.678 | 0.632 | 0.303 | 0.418 | 0 | 0.278 | 0.394 | 0.495 |
| **CD130** | 27.2 | 3.92 | 6.03 | 3.38 | 2.46 | 1.16 | 5.59 | 5.37 | 4.25 | 6.22 |
| **CD134** | 0.927 | 0.76 | 0.639 | 0.598 | 0.126 | 0.725 | 0.557 | 0.0518 | 0.197 | 0.403 |
| **CD135** | 0.745 | 0.837 | 0.787 | 0.423 | 0.0952 | 0.477 | 0 | 0.37 | 0.189 | 0.229 |
| **CD137** | 0.929 | 6.84 | 3.24 | 1.48 | 0.947 | 0.467 | 2.08 | 1.28 | 0.533 | 2.05 |
| **CD137L** | 0.539 | 0.757 | 0.795 | 0.471 | 0.27 | 0.372 | 0.546 | 0.212 | 0.363 | 0.447 |
| **CD138** | 0.674 | 0.811 | 0.563 | 0.45 | 0.338 | 0.454 | 5.03 | 0.0532 | 4.8 | 0.612 |
| **CD140b** | 99 | 89.3 | 98.9 | 71.8 | 86.5 | 76.1 | 57.5 | 50.2 | 78.8 | 54.2 |
| **CD141** | 6.29 | 2.51 | 20.6 | 3.01 | 9.35 | 4.16 | 1.72 | 5.71 | 0.186 | 1.01 |
| **CD142** | 38.5 | 19.6 | 49.9 | 13.5 | 24.4 | 19 | 69.5 | 25.7 | 86.6 | 0.598 |
| **CD144** | 0.649 | 0.63 | 0.73 | 0.638 | 0.0874 | 0.512 | 0.313 | 0.21 | 0.208 | 0.362 |
| **CD146** | 83.9 | 78 | 85.5 | 66.9 | 95.7 | 62.9 | 74.9 | 88.2 | 79.5 | 89.6 |
| **CD147** | 98.1 | 94.4 | 98.3 | 94.6 | 99.7 | 92.2 | 100 | 96.2 | 96.2 | 93.9 |
| **CD150** | 0.849 | 0.933 | 1.03 | 0.382 | 0.3 | 0.408 | 0.44 | 0.162 | 0.529 | 0.243 |
| **CD151** | 97.1 | 89.1 | 97.2 | 78.6 | 84.4 | 60.6 | 64.8 | 65.6 | 57.5 | 94.7 |
| **CD152** | 0.83 | 1.05 | 0.706 | 0.594 | 0.299 | 0.44 | 0 | 0.054 | 0.174 | 0.212 |
| **CD153** | 1.26 | 0.826 | 0.636 | 0.444 | 0.22 | 0.34 | 0.346 | 0.0571 | 0.949 | 0.337 |
| **CD154** | 0.607 | 0.556 | 0.751 | 0.43 | 0.246 | 0.609 | 0.162 | 0.104 | 0.189 | 0.317 |
| **CD158a** | 0.706 | 0.76 | 0.949 | 0.568 | 0.215 | 0.522 | 0.141 | 0.155 | 0.377 | 0.405 |
| **CD158b** | 0.404 | 0.49 | 0.409 | 0.403 | 0.214 | 0.377 | 0.48 | 0.346 | 0.375 | 0.178 |
| **CD161** | 0.347 | 0.434 | 0.668 | 0.224 | 0.206 | 0.387 | 0.404 | 0.0948 | 0.18 | 0.275 |
| **CD162** | 0.393 | 0.298 | 0.0931 | 0.532 | 0.212 | 0.482 | 0.162 | 0.259 | 0.19 | 0.51 |
| **CD163** | 0.665 | 0.714 | 1.2 | 0.625 | 0.147 | 0.514 | 0.39 | 0.0499 | 0 | 0.253 |
| **CD164** | 99 | 90.6 | 98.5 | 92.9 | 98.4 | 93.2 | 90.8 | 96.3 | 93.7 | 88.1 |
| **CD165** | 83.2 | 73.4 | 86 | 48.4 | 90.8 | 41.3 | 64.3 | 87.4 | 67.6 | 26.6 |
| **CD166** | 96.5 | 83.4 | 88.6 | 88.6 | 87.5 | 63.9 | 90 | 87.8 | 79.2 | 83.9 |
| **CD171** | 3.56 | 0.824 | 0.945 | 1.03 | 0.461 | 0.229 | 2.74 | 0.324 | 3.23 | 0.255 |
| **CD172b** | 0.881 | 0.882 | 0.597 | 0.218 | 0.0486 | 0.297 | 0.134 | 0.0525 | 0.182 | 0.176 |
| **CD177** | 0.901 | 1.21 | 0.988 | 0.448 | 0.1 | 0.356 | 0.432 | 0.165 | 0.178 | 0.431 |
| **CD178** | 0.715 | 0.497 | 0.526 | 0.523 | 0.195 | 0.363 | 0.161 | 0.102 | 0.37 | 0.369 |
| **CD180** | 0.454 | 0.947 | 0.665 | 0.563 | 0.247 | 0.409 | 0.136 | 0.225 | 0 | 0.403 |
| **CD181** | 0.7 | 0.452 | 0.627 | 0.672 | 0.362 | 0.378 | 0.92 | 0.106 | 0.372 | 0.51 |
| **CD183** | 0.909 | 0.366 | 0.742 | 0.829 | 0.186 | 0.487 | 0.308 | 0.154 | 0.187 | 0.219 |
| **CD184** | 0.464 | 0.504 | 0.54 | 0.522 | 0.375 | 0.325 | 0.462 | 0.197 | 0.375 | 0.388 |
| **CD193** | 1.02 | 0.863 | 0.709 | 0.878 | 0.218 | 0.45 | 0.415 | 0 | 0.187 | 0.535 |
| **CD195** | 1.13 | 1.4 | 1.42 | 0.586 | 0.166 | 0.521 | 0.524 | 0.0504 | 0.182 | 0.319 |
| **CD196** | 1.25 | 1.32 | 1.29 | 0.641 | 0.202 | 0.534 | 0.431 | 0.111 | 0.503 | 0.257 |
| **CD197** | 1.14 | 1.13 | 1.24 | 0.64 | 0.262 | 0.637 | 0.634 | 0 | 1.42 | 0.405 |
| **CD200** | 7.78 | 7.12 | 5.77 | 0.541 | 1.23 | 0.914 | 0.603 | 0.223 | 0.202 | 21.2 |
| **CD205** | 1.17 | 0.451 | 0.8 | 0.626 | 0.278 | 0.667 | 0.446 | 0.226 | 0.601 | 0.335 |
| **CD206** | 1.32 | 1.05 | 1.32 | 0.565 | 0.26 | 0.317 | 0.849 | 0.112 | 0.394 | 0.319 |
| **CD220** | 0.482 | 1.05 | 0.579 | 0.315 | 0.305 | 0.521 | 0.447 | 0.314 | 0.191 | 0.299 |
| **CD221** | 24.7 | 22.9 | 15.4 | 3.23 | 2.55 | 1.8 | 7.38 | 2.49 | 15.1 | 2.65 |
| **CD226** | 0.193 | 0.541 | 0.484 | 0.463 | 0.175 | 0.478 | 0.933 | 0.272 | 0.731 | 0.298 |
| **CD227** | 79.6 | 44.1 | 33.6 | 18.5 | 20.1 | 3.23 | 10.5 | 7.19 | 6.78 | 33.6 |
| **CD229** | 0.994 | 0.509 | 1.08 | 0.66 | 0.386 | 0.354 | 0.406 | 0.105 | 1.1 | 0.48 |
| **CD231** | 2.15 | 1.91 | 3.03 | 0.978 | 0.211 | 0.837 | 0.461 | 0.315 | 0.704 | 0.458 |
| **CD235a** | 7.31 | 1.33 | 4.79 | 6.03 | 5.98 | 3.03 | 9.34 | 4.67 | 13.8 | 0.769 |
| **CD243** | 1.82 | 2.47 | 2.02 | 0.737 | 0.384 | 0.282 | 0.558 | 0.218 | 0.536 | 0.256 |
| **CD244** | 1.03 | 1.3 | 0.968 | 0.618 | 0.175 | 0.496 | 0.591 | 0 | 0.563 | 0.324 |
| **CD255** | 1.15 | 2.95 | 1.29 | 1.91 | 0.207 | 0.639 | 1.13 | 0.161 | 0.427 | 2.97 |
| **CD268** | 0.718 | 0.52 | 0.792 | 0.659 | 0.29 | 0.38 | 0.525 | 0 | 0.419 | 0.222 |
| **CD271** | 2.85 | 1.24 | 1.13 | 0.756 | 0.255 | 0.625 | 0.696 | 0.271 | 3.11 | 0.765 |
| **CD273** | 7.11 | 14 | 13 | 7.56 | 7.01 | 10.5 | 24.4 | 17.3 | 42.3 | 15.6 |
| **CD274** | 20.8 | 28.9 | 19.8 | 18.4 | 20.5 | 17.5 | 62.2 | 47.3 | 57.3 | 5.84 |
| **CD275** | 0.433 | 0.447 | 0.535 | 0.654 | 0.336 | 0.737 | 0.467 | 0.161 | 0.735 | 0.357 |
| **CD278** | 0.423 | 0.349 | 0.271 | 0.545 | 0.274 | 0.525 | 0.602 | 0.107 | 1.08 | 0.252 |
| **CD279** | 0.796 | 0.775 | 0.701 | 0.53 | 0.184 | 0.526 | 0.591 | 0 | 0.362 | 0.326 |
| **CD282** | 1.51 | 0.993 | 0.983 | 0.699 | 0.348 | 0.528 | 0.562 | 0.0502 | 0 | 0.461 |
| **CD305** | 3.06 | 2.41 | 1.88 | 0.723 | 0.302 | 0.588 | 0.399 | 0.163 | 0.182 | 0.702 |
| **CD309** | 1.18 | 1.43 | 1.23 | 0.441 | 0.275 | 0.817 | 0.867 | 0.275 | 0 | 0.269 |
| **CD314** | 1.09 | 0.614 | 0.729 | 0.493 | 0.27 | 0.521 | 0.547 | 0.112 | 0.196 | 0.424 |
| **CD321** | 46.9 | 7.95 | 43.6 | 7.4 | 8.83 | 19.2 | 19.3 | 4.76 | 21 | 0.644 |
| **CDw327** | 0.619 | 0.482 | 0.597 | 0.616 | 0.172 | 0.38 | 0.292 | 0.279 | 0 | 0.551 |
| **CDw328** | 1.35 | 0.887 | 1.36 | 0.539 | 0.272 | 0.37 | 0.764 | 0.251 | 0.344 | 0.354 |
| **CDw329** | 1.46 | 1.08 | 1.37 | 0.589 | 0.382 | 0.39 | 0.708 | 0.324 | 0.748 | 0.339 |
| **CD335** | 1.1 | 0.81 | 1.41 | 0.513 | 0.593 | 0.27 | 0.797 | 0.306 | 1.26 | 0.342 |
| **CD336** | 0.917 | 1.05 | 0.784 | 0.502 | 1.24 | 0.584 | 1.29 | 1.19 | 1.06 | 0.363 |
| **CD337** | 1.31 | 1.08 | 0.986 | 0.416 | 0.299 | 0.512 | 0.882 | 0.226 | 0 | 0.391 |
| **CD338** | 3.73 | 1.05 | 1.54 | 0.868 | 0.227 | 0.376 | 0 | 0.113 | 0 | 0.585 |
| **CD340** | 94.6 | 56.3 | 76.5 | 37.8 | 25.6 | 25.2 | 24.9 | 13.8 | 26 | 33.3 |
| **abTCR** | 1.06 | 0.95 | 0.749 | 0.699 | 0.258 | 0.448 | 0.311 | 0.171 | 0.203 | 0.345 |
| **B2-uGlob** | 95.7 | 93.3 | 98 | 88.6 | 85.5 | 68.7 | 90.6 | 81.2 | 79.4 | 90.2 |
| **BLTR-1** | 0.371 | 0.62 | 0.788 | 0.534 | 0.204 | 0.325 | 0.437 | 0 | 0.39 | 0.152 |
| **CLIP** | 0.915 | 0.618 | 0.951 | 0.657 | 0.124 | 0.432 | 0.273 | 0.16 | 0 | 0.236 |
| **CMRF-44** | 1.17 | 1.22 | 1.57 | 0.512 | 0.149 | 0.697 | 0.412 | 0.328 | 0.943 | 0.205 |
| **CMRF-56** | 1.78 | 0.862 | 0.914 | 1.37 | 0.149 | 0.292 | 0.259 | 0.326 | 0.548 | 0.497 |
| **Fmlp-r** | 0.835 | 0.853 | 0.875 | 0.492 | 0.293 | 0.55 | 0.43 | 0.331 | 0.755 | 0.435 |
| **gd TCR** | 1.16 | 0.724 | 0.813 | 0.295 | 0.612 | 0.583 | 1.62 | 0.102 | 0.361 | 0.394 |
| **Hem. Prog. Cell** | 8.4 | 1.56 | 0.88 | 1.28 | 0.253 | 0.389 | 1.11 | 0.277 | 0.185 | 0.615 |
| **HLA-A,B,C** | 98.6 | 98.4 | 99.4 | 99.6 | 81.8 | 98.1 | 92.3 | 74.8 | 81.9 | 96.9 |
| **HLA-A2** | 1.34 | 0.971 | 0.865 | 0.408 | 0.65 | 0.488 | 0.665 | 0.197 | 0.348 | 0.194 |
| **HLA-DQ** | 43.9 | 43.9 | 48.3 | 34 | 5.18 | 29.3 | 8.03 | 12.6 | 6.74 | 30.1 |
| **HLA-DR** | 0.564 | 0.487 | 0.508 | 0.62 | 0.159 | 0.467 | 0.27 | 0.203 | 0.697 | 10.1 |
| **HLA-DR,DP,DO** | 1.25 | 0.836 | 0.823 | 0.553 | 0.368 | 0.401 | 0.565 | 0.171 | 1 | 11 |
| **Invariant NKT** | 0.828 | 0.592 | 0.711 | 0.53 | 0.226 | 0.724 | 0.759 | 0.0509 | 0.174 | 0.327 |
| **Disialoganglioside GD2** | 3.21 | 5.09 | 1.73 | 2.82 | 8.96 | 0.873 | 0.675 | 1.07 | 0.71 | 26.3 |
| **MIC A/B** | 5 | 0.752 | 12.6 | 17.4 | 0.517 | 16.1 | 2.92 | 0.612 | 7.5 | 0.522 |
| **NKB1** | 1.31 | 0.626 | 1.09 | 0.588 | 0.276 | 0.276 | 1.33 | 0.212 | 1.02 | 0.457 |
| **SSEA-1** | 1.14 | 0.925 | 1.21 | 1.2 | 0.414 | 2.04 | 1.62 | 1.01 | 8.1 | 0.672 |
| **SSEA-4** | 75.4 | 84 | 80.5 | 66.9 | 87.4 | 52.7 | 10.6 | 45.5 | 6.43 | 80.3 |
| **TRA-1-60** | 0.809 | 0.964 | 0.825 | 0.295 | 0.263 | 0.563 | 0.57 | 0.22 | 1.15 | 0.428 |
| **TRA-1-81** | 0.707 | 0.474 | 0.597 | 0.555 | 0.412 | 0.649 | 0.462 | 0.345 | 0 | 0.403 |
| **Vb 23** | 0.777 | 0.515 | 0.884 | 0.451 | 0.264 | 0.303 | 0.153 | 0.162 | 0.201 | 0.239 |
| **Vb 8** | 0.656 | 1.01 | 0.688 | 0.508 | 0.293 | 0.484 | 0.408 | 0.104 | 0.36 | 0.37 |
| **CD326** | 3.55 | 1.55 | 1.35 | 1.33 | 0.413 | 0.684 | 0.388 | 0.201 | 0.484 | 0.363 |
| **mIgM** | 1 | 0.591 | 0.752 | 0.465 | 0.0241 | 0.404 | 0.255 | 0 | 0 | 0.294 |
| **mIgG1** | 0.994 | 1.22 | 0.931 | 0.387 | 0.0252 | 0.446 | 0 | 0 | 0 | 0.144 |
| **mIgG2a** | 0.787 | 0.758 | 0.869 | 0.346 | 0.125 | 0.266 | 0 | 0 | 0.183 | 0.208 |
| **mIgG2b** | 1.02 | 0.552 | 0.943 | 0.191 | 0.0224 | 0.355 | 0.125 | 0 | 0.167 | 0.292 |
| **mIgG3** | 0.894 | 0.864 | 0.788 | 0.627 | 0.0721 | 0.407 | 0.404 | 0.179 | 0 | 0.161 |
| **CD49f** | 73.7 | 70.5 | 75.4 | 91.5 | 75 | 89.1 | 79.1 | 84.4 | 49.2 | 88 |
| **CD104** | 3.42 | 1.23 | 1.77 | 0.803 | 0.271 | 0.319 | 0.826 | 0.27 | 0 | 0.382 |
| **CD120b** | 0.352 | 0.648 | 0.113 | 0.516 | 0.157 | 0.361 | 0.59 | 0.0511 | 0.521 | 1.73 |
| **CD132** | 0.336 | 0.269 | 0.594 | 0.86 | 0.417 | 0.756 | 1.33 | 0.207 | 0.898 | 0.596 |
| **CD201** | 94.5 | 79.1 | 95 | 96.5 | 77 | 93.8 | 50.3 | 53.4 | 39.7 | 85.3 |
| **CD210** | 0.312 | 0.389 | 0.214 | 0.677 | 0.184 | 0.539 | 0.485 | 0 | 0.173 | 0.283 |
| **CD212** | 0.12 | 0.249 | 0.292 | 0.645 | 0.0235 | 0.42 | 0.362 | 0.255 | 0.192 | 0.287 |
| **CD267** | 0.353 | 0.138 | 0.222 | 0.45 | 0.0463 | 0.455 | 0.528 | 0.108 | 0.366 | 0.319 |
| **CD294** | 0.515 | 0.414 | 0.495 | 0.526 | 0.146 | 0.695 | 0.214 | 0.0964 | 0 | 0.486 |
| **SSEA3** | 0.168 | 0.627 | 0.381 | 0.857 | 0.215 | 0.834 | 0.733 | 0.168 | 0.746 | 33.9 |
| **Cut. Lymph. Ant.** | 0.464 | 1.3 | 2.17 | 1.03 | 0.972 | 4.53 | 0.84 | 1.93 | 0.18 | 0.57 |
| **INT B7** | 0.39 | 0.621 | 0.457 | 0.825 | 0.0665 | 0.751 | 0.46 | 0.158 | 0.5 | 1.32 |
| **rIgM** | 0.541 | 0.212 | 0.445 | 0.573 | 0.143 | 0.61 | 0.632 | 0.108 | 0 | 0.319 |
| **rIgG1** | 0.337 | 0.261 | 0.64 | 0.323 | 0.146 | 0.67 | 0.26 | 0.169 | 0.2 | 0.52 |
| **rIgG2a** | 0.403 | 0.192 | 0.111 | 0.364 | 0.26 | 0.448 | 0.535 | 0.218 | 0.358 | 0.214 |
| **rIgG2b** | 0.248 | 0.421 | 0.16 | 0.558 | 0.182 | 0.525 | 0.259 | 0.259 | 0.172 | 0.207 |

**Supplementary Methods**

**Derivation and characterization of human iMSC lines**

Skin biopsies were collected from healthy individuals after written informed consent. Human iPSC lines were derived, quality controlled, expanded, and characterized using the Global Stem Cell Array® as previously reported ^1^. Generated iPSC lines were expanded in StemFlex™ medium (Thermo Fisher Scientific) on Geltrex™-coated plasticware before mesenchymal induction (iPSC 🡪 iMSC). The expression of pluripotency makers was confirmed via immunofluorescence. Briefly, cells were fixed in 4% paraformaldehyde, permeabilized with 0.1% Triton X-100 in PBS, blocked with 5% (vol/vol) donkey serum (Jackson ImmunoResearch Laboratories Inc.) in PBS, and incubated overnight with primary antibodies against TRA-1-60 (2 µL/mL; Stemgent, 09-0010) and Oct-4 (2 µL/mL; Stemgent, 09-0023). Alexa Fluor secondary antibodies were used for detection (1 µL/mL; Invitrogen, A32795 and A32773). Colonies were counterstained with phalloidin (25 µL/mL; Invitrogen, A12379) and Hoechst 33342 (4 µL/mL; Invitrogen; H3570) to visualize the actin filaments and nuclei, respectively. Samples were imaged with an Olympus IX71 mounted with Q-Color 3 imaging camera and equipped with Olympus DP-BSW software.

For mesenchymal induction, confluent iPSC cultures were treated with induction medium consisting of KO-DMEM supplemented with 20% (vol/vol) HyClone FBS, 2 mM GlutaMAX, 0.1 mM nonessential amino acids, 0.1 mM β-mercaptoethanol, and 100 U/mL Anti-Anti (Gibco^TM)^ for 1 week. Following induction, cells were passaged using trypsin (0.25%) and cultured in gelatin-coated tissue culture flasks until they became homogenous for a fibroblastic-like morphology ^2^. Cells were expanded under similar conditions for 10 passages to investigate their proliferation potential. Briefly, cells were seeded at a density of 5,000 cells/cm^2^ and cultured in expansion medium for 5 days. At the end of each passage, cells were detached using trypsin (0.25%) and analyzed using a nucleocounter to count and measure the cell size. Results are expressed as cumulative growth.

Presence of pluripotency markers was confirmed for parental iPSC lines (Supplementary Fig. S1) and absence of these markers was also evaluated for resultant iMSC lines (Supplementary Fig. S2a). At passage 2, all iMSC lines were also karyotyped to assess genome integrity (Cell Line Genetics) (Supplementary Fig. S2b). The global surface markers profile was probed using the BD Lyoplate^TM^ Human Cell Surface Marker Screening Panel (BD Biosceinces). Briefly, cells at passage 2, 5, and 10 were detached using trypsin, washed with PBS, and barcoded with Live/Dead Fixable Dyes (Thermo Scientific; L34955, L34959, L23101, L10119) at 5x concentration. Cells were combined and incubated with BD Human Cell Surface Marker Screening Panel (BD Biosciences; 560747).  Flow Cytometry data were acquired on a 4-laser Attune NxT (405nm, 488nm, 561nm) with Autosampler. Analysis of data was performed with Flowjo v9.

The mesodermal differentiation potential was evaluated in monolayer and micromass pellet cultures. For monolayer cultures, cells at passage 5 were plated into gelatin-coated cell culture plates and cultured for 4 weeks in OsteoLife™ and AdipoLife™ media (LifeLine). Osteogenesis was evaluated by von Kossa staining of calcium deposition following standard procedures. Adipogenesis was assessed by Oil Red O staining of accumulated lipids following standard procedures. For micromass pellet cultures, 500,000 cells at passage 5 were centrifuged at 300 × g for 5 minutes and cultured for 4 weeks in ChondroLife™ medium (LifeLine). Next, pellets were fixed in 4% paraformaldehyde, washed, cryopreserved in 30% w/v sucrose, and finally embedded in optimal cutting temperature (OCT) solution (Sakura) in isopentane. Thereafter, samples were sectioned using a cryostat at -21 °C and transferred to glass slides (Superfrost plus) for staining. The presence of glycosaminoglycans (GAG) was evaluated by Alcian blue (LifeLine) stain using standard procedures. Expansion medium was used as control for all differentiation experiments.

**Macromer synthesis**

HA was converted to its tetrabutylammonium salt (HA-TBA) so that it would be soluble in dimethyl sulfoxide (DMSO). To make HA-TBA, sodium hyaluronate (NaHA, LifeCore, 60 kDa) was dissolved in deionized water (DI H_2_O) at 2 wt% and the Dowex 50WX2 200-400 mesh proton exchange resin was added to the solution (3 g resin per 1 g NaHA) and allowed to exchange for 2 h. The resin was filtered off and the pH of the filtrate was increased to 7.02 - 7.05. The resulting solution was frozen at -80^O^C, lyophilized, and stored at -20^O^C until used. Figure S5A shows ^1^H NMR spectrum of HA-TBA.

To form biofunctionalized macromers (HANorMe-cRGD), a three-step approach was performed (Supplementary Fig. S4b). The carboxylic acid residues of hyaluronic acid (HA) were first modified with norbornene (HANor) using BOP coupling chemistry as previously reported ^3^. Next, the hydroxyl residues of HANor were modified with methacrylate groups (HANor-Me) ^4^. To biofunctionalize HANor-Me, macromers were mixed with thiol-containing peptides resulting in a Michael addition reaction between methacrylates in HANor-Me and thiols (via cysteine “C” residues) on peptides. Using this scheme, thiolated RGD peptides (sequence: G**C**GYGRGDSPG) were coupled to HANor-Me macromers (HANor-Me-cRGD) to permit cell attachment. To form hydrogels with RGD adhesive motifs, HANor-Me-cRGD macromers were crosslinked with dithiothreitol (DTT) crosslinkers via a thiol-norbornene click reaction to form hydrogels. To our knowledge this is the first study that coupled both norbornene and methacrylate groups to the HA backbone to allow for crosslinking of peptide functionalized macromers. Macromer modification was confirmed through ^1^H NMR (Supplementary Fig. S5). HANor-Me-cRGD macromers (3 wt%) were mixed with a low (0.5 mM), medium (1.0 mM), or high (2.0 mM) concentration of DTT crosslinker and photopolymerized with ultraviolet (UV) light (10 mW/cm^2^, 5 min) to form hydrogels of varying stiffness. Elastic moduli of Low, Med, and High stiffness hydrogels were found to be 5.19 ± 1.04, 9.58 ± 0.98, and 19.27 ± 2.41 kPa, respectively (Supplementary Fig. S4b).

To synthesize HANor, HA-TBA was dissolved in anhydrous DMSO (2 wt%) with a 3:1 M ratio of 5-norbornene-2-methylamine to HA-TBA repeat units. Benzotriazol-1-yloxy tris(dimethylamine)phosphonium hexafluorophosphate (BOP) reagent was added by syringe at a 0.4 M ratio to HA-TBA repeat units. After 2 h, cold water was added to quench the reaction and the solution was purified by dialysis for 3 d to remove DMSO. The resulting solution was frozen at -80^O^C, lyophilized, and stored at -20^O^C. The product was analyzed by ^1^H NMR spectroscopy and the HANor was found to have ~50% of its repeat units functionalized with norbornene (Supplementary Fig. S5b).

HANorMe was synthesized by reacting HANor with methacrylic anhydride at 1 wt% in DI H_2_O at pH of 8. The solution was purified by dialysis for 3 d, frozen at -80^O^C, lyophilized, and stored at -20 ^O^C until use. The extent of methacrylation was confirmed by ^1^H NMR (Supplementary Fig. S5c). RGD peptide (1025.06 Da) with a cysteine residue at the C-terminal end was obtained from GenScript. For Michael-type addition peptide coupling, 11.49 mg of peptide and 100 mg HANor-Me were dissolved in triethanolamine-buffered saline (TEOA buffer, pH 8). This coupling ratio was used to obtain a final peptide concentration of 2 mM in a 3 wt% hydrogel. The solution was purified by dialysis for 3 d, frozen at -80^O^C, lyophilized, and stored at -20^O^C until use. The extent of cRGD coupling was confirmed by ^1^H NMR (Supplementary Fig. S5d).

**Immunofluorescence staining**

After 3 days in culture, stem cell-laden hydrogels were fixed in 10% formalin for 10 min at room temperature. Samples were then permeabilized with 0.1% Triton X-100 for 2 mins and blocked with 3% bovine serum albumin in PBS for 30 mins. Primary YAP or pFAK antibody (Santa Cruz Biotechnologies, 1:200) was added for 1 h, followed by Alexa Fluor 488 secondary antibody (Life Technologies, 1:200) for 2 h at room temperature. To visualize individual cells and nuclei, samples were stained for actin (Alexa Flour 568 phalloidin, 20 mins, 1:100) and double stranded DNA (Hoechst, 5 mins, 1:1000), respectively.

**Microscopy and image analysis**

Acquisition of immunofluorescence images was performed with a Nikon A1 confocal microscope. Morphological features (area, circularity, aspect ratio) were quantified using ImageJ (https://imageJ.nih.gov./ij/). Images of YAP staining was analyzed for nuclear YAP localization analysis by measuring the fluorescence of five regions of interest (ROI) on the cytoplasm and nucleus of each cell, respectively. The ratio between the mean fluorescence of the nuclear and cytoplasmic ROIs were defined as the nuclear YAP value. To assess focal adhesions, the major axis of stained pFAKs per cell were measured using the measurement feature in ImageJ. The number of cell adhesions per cell was determined using the Find Maxima feature in ImageJ. Actin anisotropy was quantified by determining the common directionality of actin fibers within a manually defined ROI using the FibrilTool plugin in ImageJ ^5^. Three ROIs were set over the perinuclear actin cap and dorsal actin fibers of each cell and the mean value is reported.

**Supplementary References**

1. Paull, D. *et al.* Automated, high-throughput derivation, characterization and differentiation of induced pluripotent stem cells. *Nat. Methods 2015 129* **12**, 885–892 (2015).

2. Peppo, G. M. de *et al.* Engineering bone tissue substitutes from human induced pluripotent stem cells. *Proc. Natl. Acad. Sci.* **110**, 8680–8685 (2013).

3. Gramlich, W. M., Kim, I. L. & Burdick, J. A. Synthesis and orthogonal photopatterning of hyaluronic acid hydrogels with thiol-norbornene chemistry. *Biomaterials* **34**, 9803–9811 (2013).

4. Smeds, K. A. & Grinstaff, M. W. Photocrosslinkable polysaccharides for in situ hydrogel formation. (2000) doi:10.1002/1097-4636.

5. Boudaoud, A. *et al.* FibrilTool, an ImageJ plug-in to quantify fibrillar structures in raw microscopy images. *Nat. Protoc.* **9**, 457–463 (2014).
